# Leveraging an intelligent slug flow platform for self-optimization of reaction systems with categorical variables†

**DOI:** 10.1039/d5sc04715c

**Published:** 2025-10-13

**Authors:** Florian L. Wagner, Gernot Neun, Thomas Tampone, Zhen Lei, Frederic G. Buono, Christopher A. Hone, C. Oliver Kappe

**Affiliations:** a Center for Continuous Flow Synthesis and Processing (CCFLOW), Research Center Pharmaceutical Engineering GmbH (RCPE) Inffeldgasse 13 8010 Graz Austria christopher.hone@rcpe.at; b Institute of Chemistry, University of Graz, NAWI Graz Heinrichstrasse 28 8010 Graz Austria oliver.kappe@uni-graz.at; c Boehringer Ingelheim Pharmaceuticals, Inc 900 Ridgebury Road Ridgefield CT 06877 USA

## Abstract

In this work we describe the development of a chemistry-based encoding approach utilizing nucleophilicity to perform Bayesian optimization campaigns. A fully automated slug continuous flow platform leveraging a liquid handler to investigate categorical variables is used for the self-optimization of organic reactions. We compared our chemistry-based approach to a chemistry-agnostic label-encoding approach. The use of encoding a physical property allowed the optimization to proceed rapidly and more successfully than existing methods, identifying not only the correct discrete parameter in the system, but also favorable conditions at the same time. Reactions were analyzed using two complementary process analytical technologies (PATs), Fourier-transform infrared spectroscopy (FT-IR) and ultra high performance liquid chromatography (UHPLC). This approach was applied to two different nucleophile-catalyzed amide coupling reactions, for single and multi-objective optimization. A long run was performed as a comparison to the slug flow operation with the liquid handler-based slug flow reactor.

## Abstract 2

Using a liquid handler, categorical variables have been investigated in self-optimization. We use an autonomous closed-loop platform to demonstrate a method to encode categorical variables based on simple, physical chemistry-based relationships.

## Graphical abstract

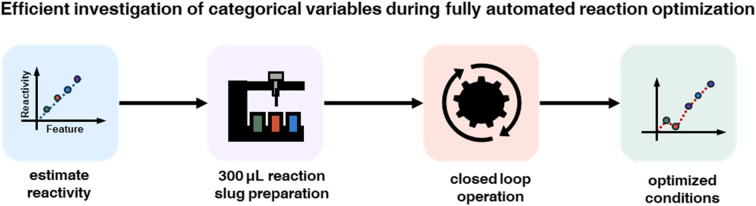

## 1 Introduction

Chemical reaction optimization is a complex and important challenge in organic chemistry.^1^ Reaction parameters greatly influence the outcome of chemical reactions. Moreover, reaction parameters often show interdependencies to each other in often complex and non-linear fashions. Reaction parameters can be continuous (numerical), for example temperature, reaction time and reagent equivalents. Alternatively, parameters can be discrete (categorial) parameters, such as the identity of solvent, base, ligand or catalyst. Reaction optimization through the investigation of one variable at a time (OVAT) is still the most common optimization approach used in organic chemistry. However, this approach is inefficient and can lead to making incorrect conclusions, as it can fail to fully capture interaction effects between parameters. Design of Experiments (DoE) is also exploited in chemical development to understand the influence of the input parameters on the process performance through the generation of a statistical design from an experimental design.^2,3^ More recently, automated self-driving systems based on optimization algorithms have been reported for the identification of optimal operating conditions.^4–7^ However, in model-based optimization approaches, it is difficult to investigate categorical parameters, as either a distinct model for each categorical parameter is constructed or the categorical parameter is assigned a numeric value on a scale, often also involving the use of principal component analysis (PCA).^8,9^

The main problem with many of the established optimization approaches used is that they tend to be relatively complex and challenging to implement, because they tend to require specialized expertise in machine learning (ML) techniques. There are several examples of published deep-learning models that use reaction data from large reaction databases,^10–12^ but this approach has several drawbacks. The first drawback is complexity, as it is not easy to construct such models for a reaction of interest. The second drawback is the data availability, even with access to large amounts of unprocessed reaction data (such as the corpus of Scifinder, Reaxys and the Open Reaction Database^13^). A further issue with utilizing published reaction data include the inherent positive bias present in the data, since published reaction data is often skewed towards more desired outcomes. Furthermore, there can be issues with reproducibility across different datasets. Reaction data are collected and reported in a non-standardized fashion and are also biased towards specific, common substrates and reactions, which complicates model building and the potential model predictive power. Another approach involves the use of PCA for dimensionality reduction.^8^ More complex approaches also exist, involving techniques derived from quantum mechanical (QM) calculations to develop reactivity models,^14,15^ but they can be challenging to implement for non-experts, both in terms of the QM simulation studies and the ML model building.

In recent years, several automated chemistry platforms have been reported as an enabling technology for screening and optimization of reactions both in a batch and flow context.^5,16–22^ High-throughput experimentation (HTE) has been adopted to rapidly screen large numbers of categorical parameters in organic synthesis,^23,24^ this is usually done by first screening the categorical parameters, then drawing conclusions about their impact. However, it is typically challenging to implement integrated process analytical technology (PAT) in such a system in an efficient manner. HTE also suffers from less precise control of process parameters, such as temperature, pressure and reaction times, which makes it more difficult to apply for a robust process optimization. In a flow chemistry system, the problems are the opposite. It is more amenable for the implementation of PAT,^25^ but varying a categorical parameter is very challenging, due to having fixed feed solutions which are typically difficult to interchange and prepare “on-the-fly”. Another issue in investigating categorical parameters in flow is the risk of reaction clogging due to unexpected solid formation. The most common approach to performing closed-loop self-optimization incorporating categorical variables is by using a liquid handler and a slug/droplet flow regime.^26–29^ In this method the reactants are separated from the solvent stream using an immiscible medium, such as inert gas or perfluorinated alkanes. The liquid handler is used to prepare the feed mixture and inject into the flow system for each experiment.

Desimpel *et al.* published an example in which a slug flow platform uses a reactive gas as a separator, utilizing O_2_ gas both as a spacer to separate their reaction slug from the solvent stream and as a reactant in a photochemical synthesis of acetophenone.^30^ In this example the authors performed closed-loop self-optimization on this complex reaction system using online UHPLC as PAT and the MVMOO algorithm.^31^

Baumgartner *et al.* developed a droplet flow system in which a liquid handler prepares a reaction droplet of only 15 μL into a gas-filled heated oscillating flow reactor.^32^ Their system consists of a U-shaped tube, in which the reaction mixture is pushed back and forth by alternating the gas pressure inlet, until the required residence time is reached. Using this platform they addressed problems such as screening ligands and bases in Pd-catalyzed cross-coupling reactions,^28^ both using traditional screening methods and more complex optimization techniques such as their MINLP2 algorithm.^32^ The MINLP2 algorithm uses an iterative response-surface method to perform global optimization of the design space. This algorithm can natively handle categorical variables and can automatically refine to reject poorly performing ligands, but it is relatively expensive in terms of iterations (60 experiments), as it relies on the construction and refinement of a linear response-surface model.

Another approach currently used in closed-loop self-optimization technique is Bayesian optimization (BO).^33^ BO is useful in optimizing chemical reactions, because it can efficiently optimize expensive-to-evaluate functions, including having applications in robotics, A/B testing and neural network hyperparameter tuning.^33^ BO strategies treat the target function as a black box. Using evaluations of the target function, a cheap-to-evaluate surrogate model is fitted. This model is then used to decide the next evaluation of the target function based on certain criteria, such as the best expected result.^34^ BO has been utilized to solve many chemical problems such as the optimization of chemical reaction conditions for single step^24,35^ and telescoped reactions,^6,36,37^ extraction processes^38^ and HPLC method development.^39^

BO is very suitable for optimization problems that mainly consist of continuous parameters, but optimization problems in chemistry also involve many categorical variables. These categorical variables are often of crucial importance, but due to the challenges in implementing them into self-optimization methods these are often overlooked or poorly addressed in automated optimization studies.

**Fig. 1:**
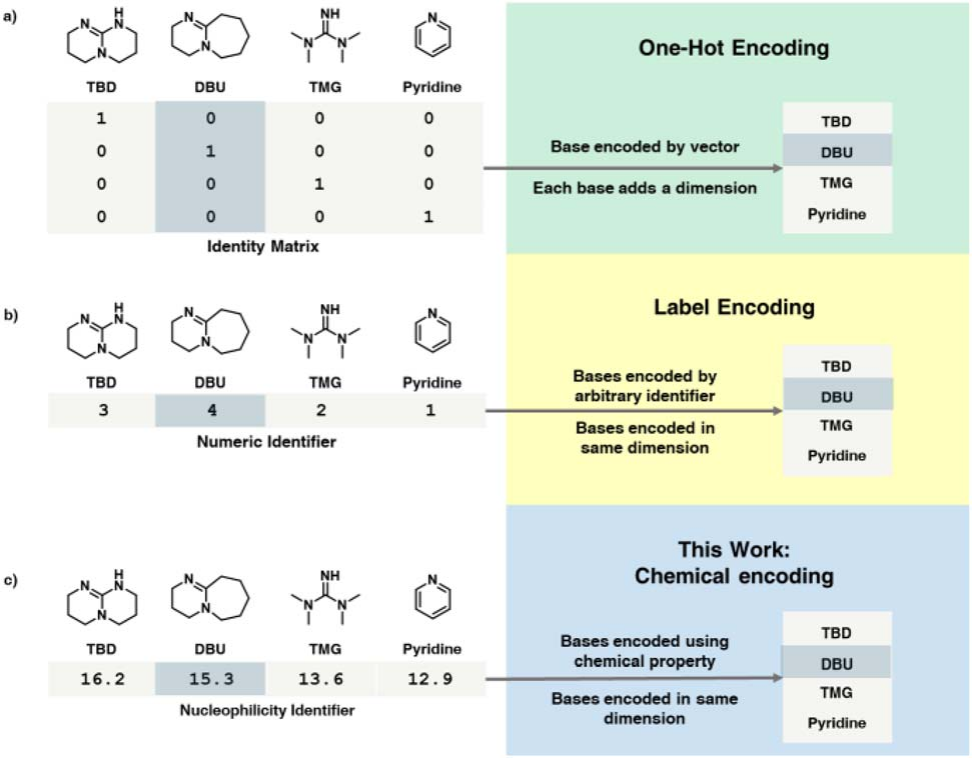
Comparison of one-hot encoding (a), label encoding (b) and this work (c) which encodes reagents based on literature-derived physical chemistry parameters.

Another key issue in mathematical approaches such as self-optimization is that it requires all variables to be represented in a numerical fashion. There are two main approaches to resolving this problem. Either a pre-optimization process is necessary to decide and fix the categorical parameters in advance (removing them from the problem posed to the algorithm) or utilizing an encoding process (Fig. 1) to handle categorical variables, converting them from discrete entities to numerical values. The most common approach in chemistry thus far has been one-hot encoding (OHE).^24,40–42^ Using this approach, the choice of categorical variable is represented as a column/row vector of an identity matrix. This simple approach separates the different choices in an orthogonal fashion but also increases the number of dimensions by the number of choices. Another approach to encoding categorical variables is label encoding.^1^ In this approach, the individual variables are assigned a numerical label and selected based on that identifying number. This strategy avoids the increase in dimensionality caused by OHE, but simultaneously introduces new, often arbitrary relationships between the different choices. One problem with both encoding methods is that they do not account for any intrinsic chemical property of the categorical parameters themselves. Other more advanced approaches used in chemistry are approaches based on structural molecular descriptors^43^ and DFT-based featurization,^24^ but as discussed previously, such approaches suffer from high complexity and have challenges associated with obtaining and handling the data, as they often require domain knowledge of theoretical chemistry and statistical methods. In addition, despite this added complexity, in some reported examples these highly-complex encoding methods are outperformed by OHE.^24,40^

In this work we propose a simple chemistry-based encoding method: by relating widely available physical chemistry based descriptor to reaction performance. There are many available descriptors, such as p*K*_a_ of reactants, solvent polarity or ligand cone angle. In this work we relate Mayr's nucleophilicity parameter (*N*) to the reactivity.^44^ This parameter is then used to encode categorical variables for use in self-optimization and model-building. These parameters directly relate to chemical reactivity *via* reaction kinetics, unlike solely data-driven and empirical approaches. This strategy will help to accelerate reaction optimization, particularly when dealing with many categorical variables and offers a good compromise between the benefits of automated self-optimization, even at an early stage with a low experimental budget available.

## 2 Results and discussion

### 2.1. Preliminary simulation studies

**Fig. 2:**
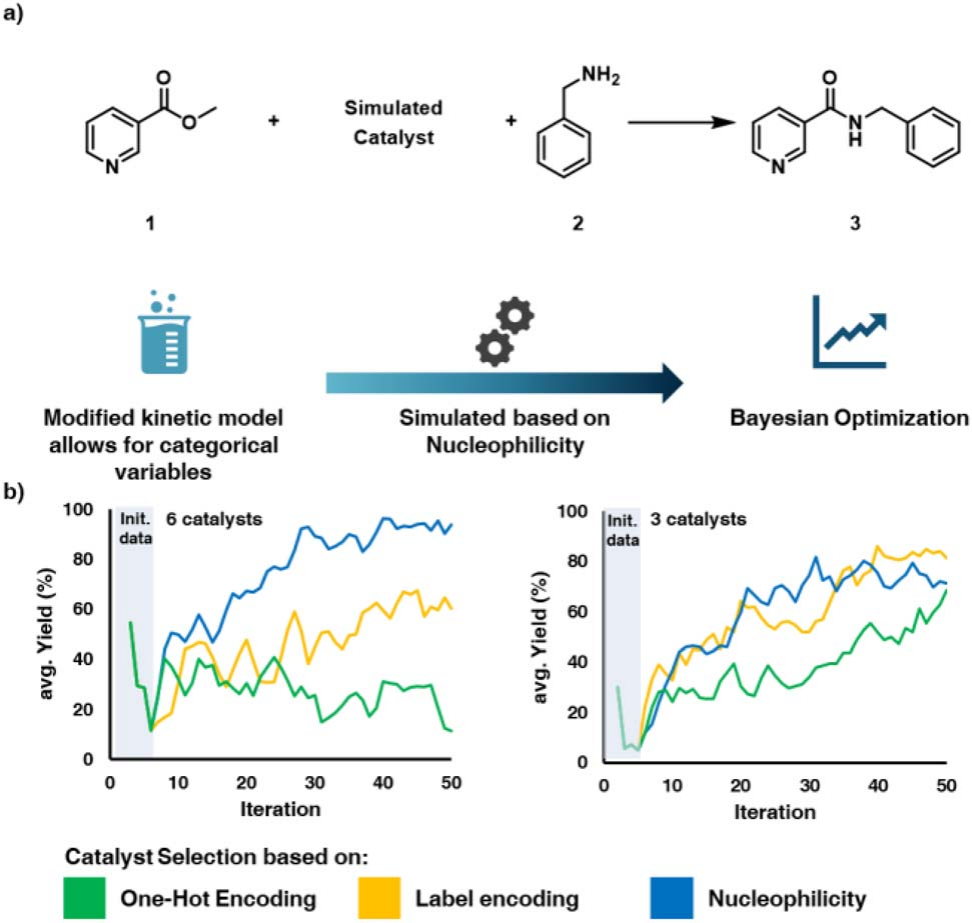
(a) Setup of simulated optimization study to investigate viability of approach (b) comparison of different encoding methods for 3 and 6 simulated catalysts.

To test the viability of this approach we performed *in silico* optimization to compare this chemical encoding approach to label encoding and one hot encoding. To this end, a simulation strategy was developed (Fig. 2a), which was based on a previously reported kinetic model of the 1,5,7-triazabicyclo[4.4.0]dec-5-en (TBD)-catalysed amidation of methyl nicotinate (1) with benzylamine.^45^ This model was modified to allow for the simulation of different catalyst types, where their relative reactivity in the model was derived from the nucleophilicity, which is available from Mayr's Database of Reactivity Parameters.^46^ Details on the modified kinetic model can be found in the SI. The kinetic model was used to simulate a series of Bayesian optimization campaigns using the BO library Summit,^47^ varying five continuous parameters (temperature (10–200 °C), reaction time (0.5–5 min), concentration of 1 (0.1–0.3 M), amine 2 equivalents (0.5–1.5 eq.) and equivalents of catalyst (0.05–5 eq.)) and one categorical parameter (catalyst type). Three different encoding methods (OHE, label encoding and chemistry-based encoding) were compared by running simulated optimization campaigns using the Thompson-sampling efficient multi-objective optimization (TS-EMO) algorithm.^48^ The TS-EMO optimization algorithm utilizes a process known as Thompson sampling in its acquisition function. Thompson sampling is a heuristic technique that involves randomly sampling the posterior distribution of the underlying Gaussian Process (GP) model. This random sampling enables the algorithm to balance exploration and exploitation. In TS-EMO specifically, there also exist considerations for the multi-objective optimization case in the form of sampling a large number of functions from the underlying GP model and refining that sample using a genetic algorithm (NSGAD-II).

Six different simulated catalysts were considered in the first simulation study (Fig. 2b). In the case of the label encoding case, the order or the catalysts was randomized, to avoid providing an implied order of reactivity to the optimization algorithm. The experimental budget was fixed at 50 iterations. In the case with only 3 catalysts to choose from both of the dimensionally reduced strategies (label encoding and nucleophilicity encoding) improve at a similar rate, converging towards an optimum after 30 to 40 iterations. OHE performs poorer on average, with high yielding results appearing less consistently throughout the optimization process, never converging to any specific value. This result suggests that the dimensionality reduction offered by label-based approaches has a favourable impact on the optimization process, even if the number of categorical choices is relatively low. Next, the TS-EMO algorithm was used to optimize a larger set of six simulated catalysts. In this example, a significant difference in performance between the encoding methods could be observed, with the chemistry-based encoding outperforming both label encoding and OHE. Label encoding improved at a slower rate. The TSEMO algorithm does not natively handle categorical variables and discontinuous search-spaces well, as shown in Fig. 2b with the one-hot encoding example, therefore a different encoding method needed to be used. Label encoding is implemented using a Euclidian distance approach, the closest label to the algorithm suggestion is selected and used. The chemical encoding uses a similar distance-based labelling approach, but the order and distance between the discrete variables is determined by the relevant chemical property of the catalysts.

These studies were also repeated utilizing the simpler single-objective Bayesian optimization (SOBO) algorithm provided in the Summit package. This algorithm utilizes a different acquisition function, expected improvement (EI). EI considers the confidence bounds of the Gaussian process model, choosing points based on the biggest numerical improvement, while balancing it with the probability of improvement as well. This algorithm is much more exploitation focused than TS-EMO. This characteristic is generally a quality we consider to be less desirable in chemical reaction optimization, as seeing many different conditions is more interesting than refining the same set of conditions, especially with a low experimental budget. The performance of this algorithm in the *in silico* study is similar between label encoding and chemistry-based encoding, but still significantly worse for the OHE case, more information on the SOBO simulations can be found in the SI.

### 2.2. Preliminary batch experiments

A model reaction was then selected to demonstrate the chemistry-encoding method experimentally (Fig. 4a), the catalytic amidation of ethyl cyanoacetate (4) using piperidine (5). This reaction also serves as a model for the synthesis of a fragment in tofacitinib (a JAK inhibitor), an active pharmaceutical ingredient (API).^49^ Initially, preliminary batch experiments were carried out with a fixed set of reaction conditions (1 eq. piperidine (5), 0.25 M ethyl cyanoacetate (4), 0.2 equiv. catalyst, 30 minutes of reaction time and a temperature of 70 °C), varying only the nucleophilic catalyst to determine the impact of the catalyst on the reaction outcome. We deliberately selected values with the intention of not giving very low or high yields to enable the different catalysts to be compared. OTG was not present in the nucleophilicity database, therefore the nucleophilicity parameter was estimated based on linear regression from the preliminary experiments (Fig. 4c). The results were consistent with the expectation that higher nucleophilicity provides higher yields in this reaction, TBD being the most effective catalyst in this reaction, obtaining a yield of 47% (Fig. 4b). 1,8-Diazabicyclo(5.4.0)undec-7-ene (DBU) and the other guanidine bases performed slightly poorer (DBU: 35%, OTG: 32% and TMG: 20%) and pyridine was the least active catalyst of the set. A strong correlation (Fig. 4c) between the performance of the different bases and their respective nucleophilicity could be observed. The promising results of the preliminary batch experiments made us confident to further develop our slug flow platform for the self-optimization campaigns containing a categorical variable.

### 2.3. Platform and approach

**Fig. 3:**
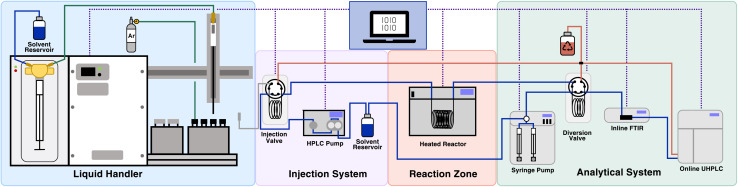
Schematic of the flow setup used in the self-optimization studies, consisting of liquid handler, injection system, a heated coil reactor and a separate flow system is used for analytics. All devices are controlled using the computer and analytical data is processed automatically to enable closed-loop optimization.

To carry out automated self-optimization experiments a slug flow platform was developed, comprising of a liquid handler (Fig. 3) to prepare the input feed. The liquid handler takes specified quantities from different vials containing the individual reaction components using a syringe. This setup allows for the reactants to be varied when preparing the reaction mixture, enabling reaction optimization involving categorical variables. This reactor platform was adapted based on one previously reported by our group.^50^ In this previous setup, a number of HPLC pumps and an automated VICI valve were used to form the feed for the reaction, this enabled the investigation of kinetics and rapid reactions that proceed at room temperature. However, it was not possible to investigate categorical variables with this system. Thus, we adapted this system incorporating a liquid-handler to form reaction slugs which does allow for the variation of categorical variables. Reaction slugs formed by the liquid handler have a volume of 300 μL and consist of the reactants at the target concentrations, diluted by solvent, as well as inert gas (N_2_) bubbles at the edges. By separating the reaction slug from the carrier solvent using inert gas, dilution of the reaction mixture by the carrier solvent is avoided.

**Fig. 4:**
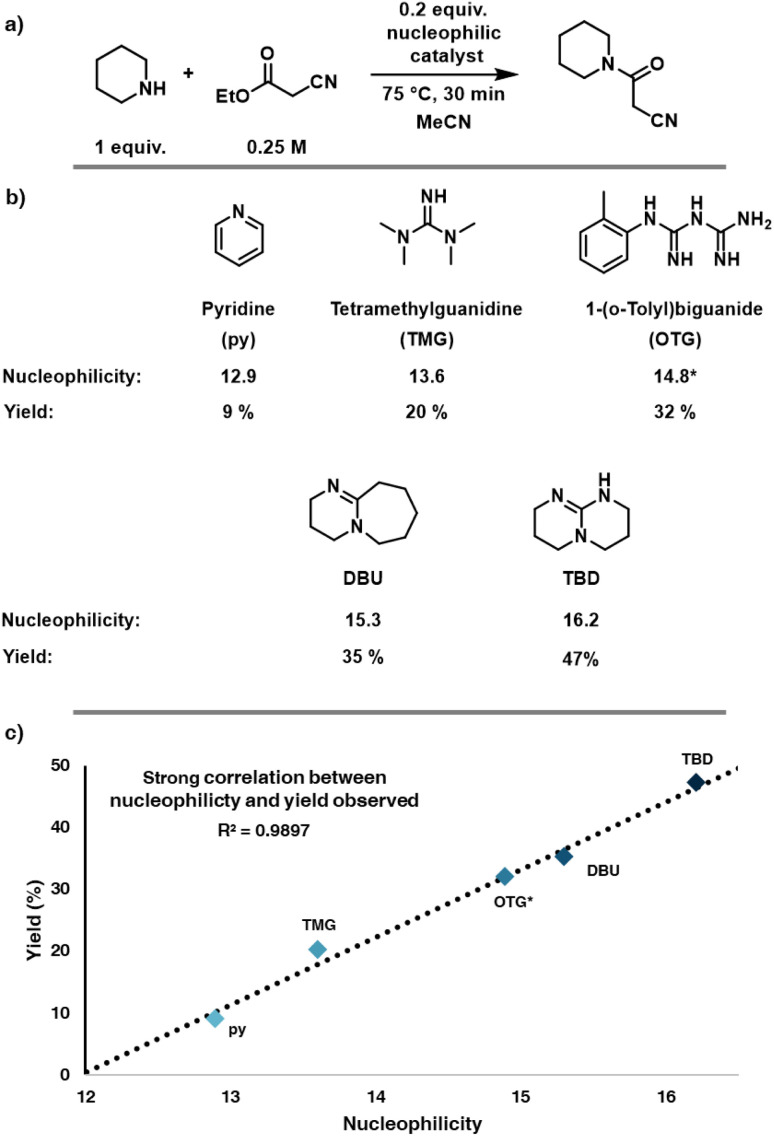
(a) Conditions of preliminary batch reactions (b) yield and nucleophilicity of preliminary batch reactions (nucleophilicity of OTG estimated based on reaction results) (c) plot of nucleophilicity against observed yield for the preliminary batch experiments.

This slug flow approach enables reactions to be performed faster, while consuming only a relatively small amount of material. The reaction slug simulates a steady-state flow experiment, while only consuming a tenth of the material required to perform the reactions. After preparing the input feed by aspirating the desired amounts from each vial of interest, the reaction mixture is injected into a sample loop and introduced into the reactor (3.15 mL) using a six-port sample injector and a carrier solvent stream (matching the reaction solvent). The mixture is then flowed through the reactor at the predetermined residence time. After the heated reaction zone, the mixture is flowed into a sample loop with an additional six-port valve, separating the reaction section from a modular analysis section containing PAT. Two PATs were utilized in tandem, FTIR to assess the consistency and reproducibility of the slug flow system and a calibrated UHPLC to determine the concentrations of the compounds of interest. The results of the slug flow system are highly reproducible (total experimental error < 5%). More information on the setup, analytics and reproducibility can be found in the supporting information. The system was utilized in a series of self-optimization campaigns.

### 2.4. Model reaction

**Fig. 5:**
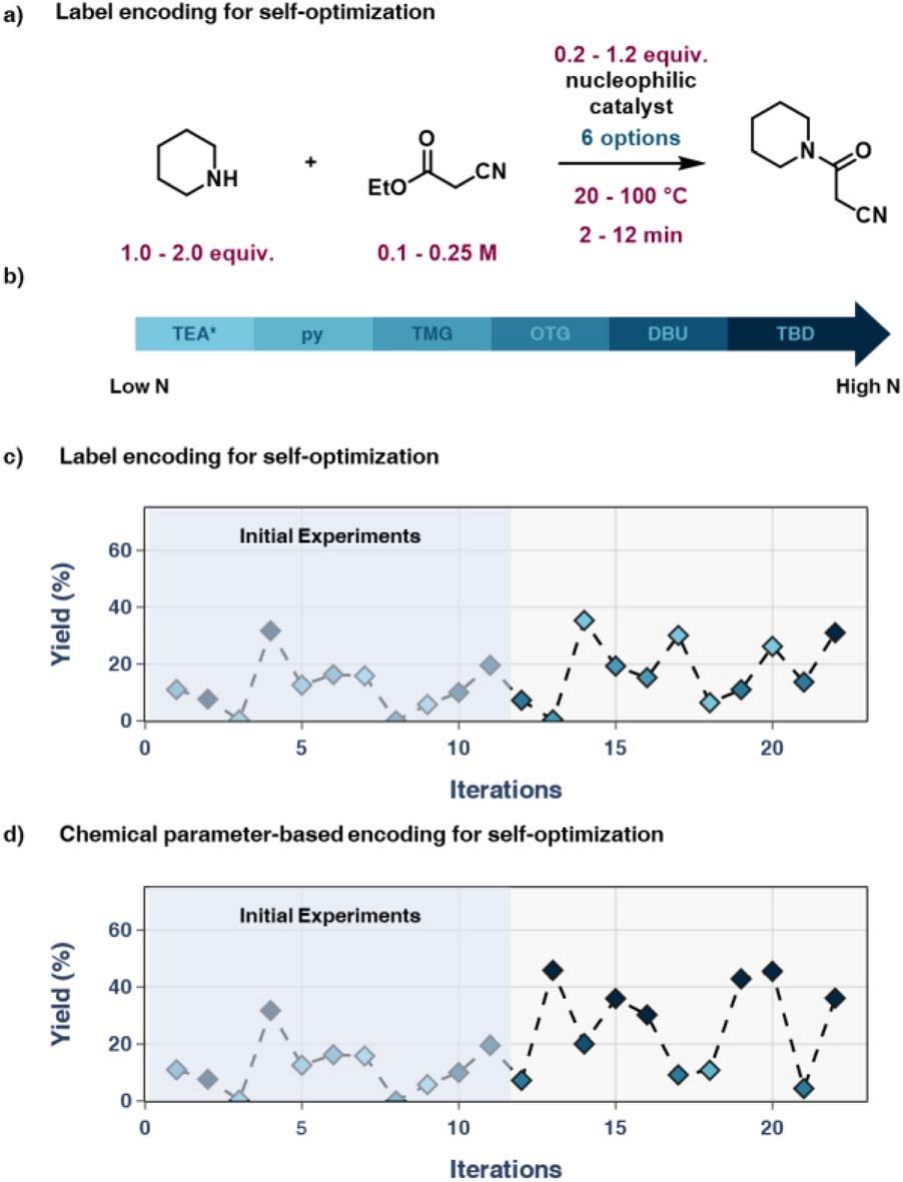
(a) Reaction and optimization ranges for self-optimization campaign. (b) List of nucleophilic catalysts used in this optimization campaign. (c) Results of self-optimization using label encoding. Most selected catalysts do not possess high nucleophilicity and results are on average lower. (d) Results of self-optimization using nucleophilicity to encode the catalysts, showing rapid improvement in results.

Finding this strong correlation between nucleophilicity and reaction outcome, a set of self-optimization campaigns was carried out with the aim of comparing the label encoding approach to the chemistry-based encoding approach. The algorithm selected was TS-EMO to allow for wider exploration of the design space. The parameters varied (Fig. 5a) were: piperidine (5) equivalents (1.0–2.0 eq.), ethyl cyanoacetate (4) concentration (0.1–0.25 M), temperature (20–100 °C), reaction time (2–12 min), catalyst equivalents (0.2–1.2 eq.) and catalyst type (6 options). With the exception of triethylamine (TEA), which was added as a 6^th^ catalyst with low expected reactivity, the catalysts (Fig. 5b) are identical to the set of bases used in the preliminary batch study. These optimization boundaries were selected to observe a range of results for conversion and yield to emphasize the impact of base selection in the optimization process. The chosen optimization parameters were: 12 initial space-filling experiments (twice the number of variables and two per base) and 10 iterations of the optimization algorithm.

In the chemistry-based encoding approach (Fig. 5d), the base favoured by the optimization algorithm was TBD with an average yield of 40%. While it can appear that the results of the label-encoding based optimization (Fig. 5c) were comparable, it is necessary to recognize that the algorithm primarily focussed on optimizing the second-most impactful variable, reaction time, while selecting bases near-randomly. All non-TBD results above 30% yield in this campaign were near the maximum possible reaction time with high equivalents of catalyst and 5. Another key factor is the speed at which the algorithms chose their relative bases, with the chemistry-informed approach primarily choosing TBD after the second experiment, while label encoding took until the 10^th^ experiment to suggest the use of TBD.

This proof-of-concept study clearly demonstrated the benefit of chemical encoding to assist the optimization algorithm in selecting the correct catalyst in this chemical transformation.

### 2.5. API example

To investigate and demonstrate the chemistry-encoding method further, an API example was investigated. Bersacapavir is an experimental drug for the treatment of hepatitis B. Medina *et al.* have developed batch processes for the synthesis of this API using a haloform amidation reaction, using both TBD and DBU.^51^ This API intermediate can be synthesized using trichloromethyl ketone 7 and aniline 8 in the presence of a nucleophilic catalyst/reagent, releasing chloroform as a leaving group. In the initial investigation developing a batch process for the synthesis of 9 by Medina *et al.* the nucleophilicity of several potential reagents was considered and a strong correlation between the nucleophilicity and the reaction outcome was observed, making this reaction an ideal candidate to serve as a complex real-life example for the approach.

**Fig. 6:**
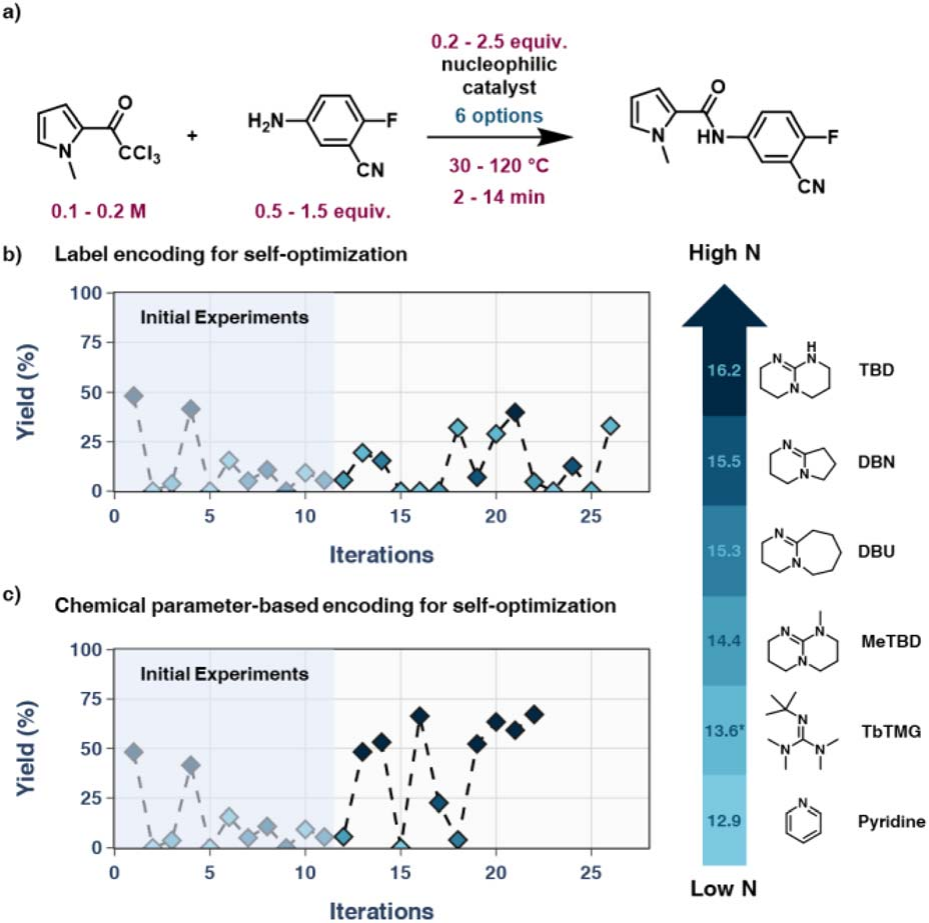
(a) Boundaries of API self-optimization example. (b) Results of label-encoding self-optimization campaign. Low yields are observed with all non-active nucleophilic catalysts and even after a 50% increase in iterations, the optimization could not identify the correct nucleophilic catalyst. (c) Nucleophilicity-based encoding self-optimization campaign. The correct catalyst is identified rapidly and yields are improved consistently.

The reaction was optimized with respect to yield as an objective using the TS-EMO algorithm within the slug flow platform, comparing a label encoding approach and a chemistry-based encoding approach. The parameters varied (Fig. 6a) for this example were: concentration of trichloromethyl ketone 7 (0.1–0.2 M), equivalents of aniline 8 (0.5–1.5 eq.), catalyst equivalents (0.2–2.5 eq.), temperature (30–120 °C), reaction time (2–14 min) and identity of the catalysts (6 options), a total of 5 continuous variables and one categorical variable, for a challenging optimization problem. For this reason, several of the catalysts were changed to more reactive compounds. TMG was replaced with TbTMG, as literature suggested that acylation of the base was the predominant reaction. OTG was replaced with MeTBD due to low amounts of product formation in the preliminary study. TEA was removed in favour of DBN, a compound similar in structure and electronics to DBU.

In this example, the label-encoded optimization campaign's experimental budget was increased by 50%. Even with this increase in iterations, the label-encoding approach was unable to identify the “correct” catalysts among the selection (Fig. 6b). As choice of catalyst is very important in this reaction, the label-encoded optimization campaign only obtained moderate yields, with no result above 50%. The disordered label encoding approach (Fig. 6b) struggled greatly and consequentially also struggled with obtaining good yields, finding no yield above 50%. TBD was only chosen once after 21 experiments; this experiment was the highest yielding result in the label-encoding based campaign at 40%. TbTMG performed significantly better than expected. It was chosen several times throughout the campaign, with an average yield of 28%, the highest among the non-TBD bases in this campaign. We hypothesize that this is due to the bulky substituent on the iminic nitrogen destabilizing the intermediate, leading to the TbTMG being eliminated more readily compared to other catalysts.

Meanwhile the chemistry-based encoding approach (Fig. 6c) favoured TBD, achieving 66% yield within only 4 algorithm-suggested experiments and similar yields were achieved in subsequent algorithm guided experiments. This much faster reaction optimization demonstrates the advantage of integrating chemical information into the Bayesian optimization process. Another interesting trend observed in this optimization was the relatively low impact of the residence time past a certain point and the need for an excess of trichloromethyl ketone 7, due to the decomposition of 7 to form the corresponding carboxylic acid. In a similar fashion, increasing the temperature improves the yield by accelerating the reaction rate towards the desired product up to a certain point. Increasing the temperature beyond 80 °C in the presence of the more nucleophilic catalysts in the optimization set appears to start reducing the yield due to the decomposition of the starting material to the acid. Unsurprisingly, increasing the equivalents of the nucleophilic catalyst beyond catalytic amounts also has a significant impact on the reaction outcome, improving the yield even for less reactive catalysts.

After these promising single-objective optimization studies, a multi-objective optimization problem was conceived to determine the impact of this chemistry-based encoding approach on a more complex optimization problem. Three objectives were considered simultaneously: maximizing the yield of the reaction, minimizing the cost of base per g of product formed and minimizing the process mass intensity (PMI),^52^ defined as the total mass of materials used/mass of product. PMI is a green chemistry metric commonly used within the pharmaceutical industry. To make the system more comparable, the obtained PMI was normalized to 1 mL. Optimizing the cost of base, the PMI and the yield is a representative problem in reaction optimization, as these objectives will be important (among others) in process development.

These three optimization objectives are competing, but they are all linked to the yield to a certain extent, pushing the algorithm to find higher yielding results. The input parameters considered in the multi-objective optimization were the same as in the single-objective yield maximization case, for a total of 6 inputs and 3 outputs. Both the PMI and cost of base per g product were assigned an upper limit.

The multi-objective case proved to be more complicated for the algorithm to optimize, as a balance between the three-objectives needed to be found. The key optimization problem here is that most of the catalysts result in relatively low yields and therefore also worse performance on the other metrics. This places additional emphasis on selecting the correct base. In the label encoding case, it took 20 iterations until TBD was selected, but this point was low-yielding within the context of the design space for the best TBD-using reaction, disincentivizing the selection of the same base afterwards. The label encoding approach began continuously improving after 8 iterations with a final result selecting TBD and finding 45% yield with good PMI and catalyst cost as well.

The chemistry-informed encoding method only selected TBD in this optimization campaign, highlighting its better performance in this reaction compared to the other options. The best point with a yield of 84% was found after only 3 iterations of the algorithm, also showing the best outcome in terms of PMI and catalyst cost. This result clearly showed the advantage of using this approach, as virtually no experiments are “wasted” on bad outcomes and good results are found rapidly. This behavior closely reflects more complex pruning-based algorithms for categorical variables, such as MINLP2.^32^

### 2.6. Steady state long run comparison

**Fig. 7:**
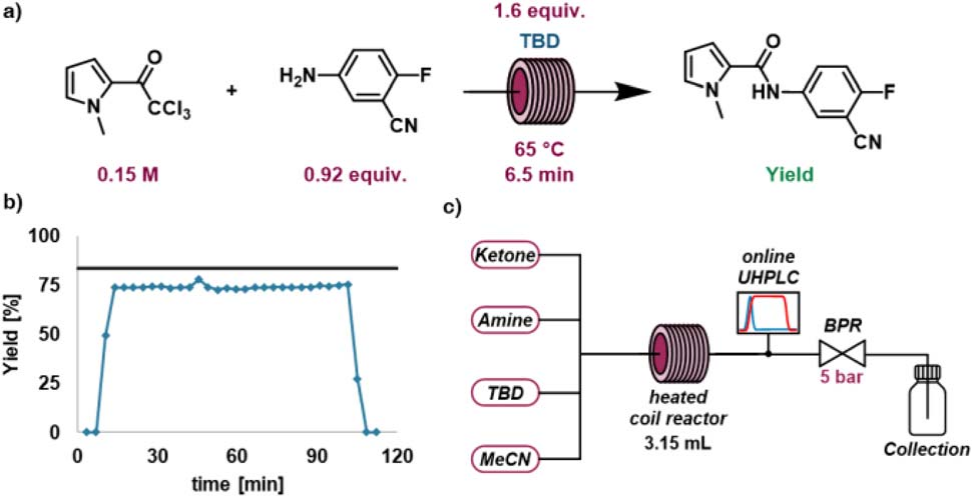
(a) Conditions of steady-state long run (b) UHPLC yield plotted against time in long-run experiment, black line marks expected yield. (c) Setup of long run experiment.

Finally, to verify the validity of the established liquid handler platform, a validation experiment was carried out by operating a long-run reaction in continuous flow. The automation platform was reconfigured from slug flow mode to continuous flow (Fig. 7c) by replacing the liquid handler with several HPLC pumps feeding reactants in a continuous fashion and bypassing the diversion loop towards the analytical instruments, directly analysing the reaction stream. The optimal experimental conditions selected during the multi-objective optimization was identified (Fig. 7a) as an attractive candidate for continuous flow operation due to performing best on all three optimization objectives. To validate the robustness of the optimized conditions the reactor was continuously operated using these setpoints. The process was operated continuously in a stable manner for 90 minutes without issues with an average yield of 74% (Fig. 7b), which is a difference of 9% from the expected yield of 83%. We subsequently experimentally validated that this difference in yields was caused by the reaction slowly occurring in the liquid handler during the droplet preparation process. It is important that users keep in mind that for liquid-handler based setups where reactions can occur at room temperature during preparation that this can slightly bias the results. In addition, we note that this is the set of conditions where the highest reaction rate would occur in the droplet process, as it provided the best results. One potential way to address this in the future would be splitting the reaction mixture into two distinct slugs and mixing them just prior to entering the reactor.

While there is a small difference in the results produced by the liquid handler and the continuous flow experiment, using this chemistry-encoding strategy the optimal base was identified quickly and efficiently while requiring only a tenth of the material ordinarily required to perform these experiments. The collected data also gives a clear indication of the relative reaction rates between the different catalysts and provides insight into the trends within the process space and very good operating conditions.

## 3 Outlook

We have shown the application of a chemistry-based encoding approach that exploits nucleophilicity for chemical encoding. In principle, any numerical chemistry descriptor, such as solvent polarity or p*K*_a_ of participating bases, could be utilized as a representation and applied in a similar fashion to encode categorical data in Bayesian optimization and machine learning applications. This approach would be especially attractive for descriptors that are easy to measure or widely accessible in databases. Potentially, the approach could also be refined further by only considering the order of the categorical parameters, instead of their discrete values. Another benefit is that utilizing this approach has some limited predictive ability, by interpolating between the relative reactivities of the different categorical parameters, the reactivity of an untested catalyst with a known nucleophilicity can be predicted and identified as a potentially promising one to test experimentally.

## 4 Conclusion

We have developed an efficient strategy for the investigation of categorical variables within an automated self-optimization platform, which leverages a liquid-handler and slug flow platform to explore the design space. The chemical encoding enabled the rapid identification of the best categorical variable and optimal conditions. This approach was compared to more established approaches, one-hot encoding and label encoding. By operating in a slug flow platform, relatively smaller quantities of material were used. The chemistry encoding approach enabled very good conditions to be identified within only 4 iterations in a fully automated closed-loop fashion.

This encoding technique was demonstrated using nucleophilicity as a chemical parameter, first in a simulation study including different number of entries in the categorical parameter and two different optimization algorithms, comparing it to label encoding and one-hot encoding methods. Automated flow experiments were then performed to demonstrate the utility of this approach for two nucleophilic amide formation reactions, including the formation of a fragment toward an API. The optimization was performed using label-encoding and the chemistry-based encoding approach. In both cases the chemistry-based encoding method outperformed the label encoding method.

## Author contributions

FLW and CAH conceived the study. FLW developed the methodology. FLW and GN performed experiments and analyzed the data. TT, ZL and FGB provided guidance to improve the quality of the experimental work and the manuscript. CAH and COK acquired funding and provided project supervision. FLW and CAH wrote the original draft of the manuscript. All authors contributed to the final manuscript.

## Conflicts of interest

There are no conflicts to declare.

## Supplementary Material

SC-OLF-D5SC04715C-s001

## Data Availability

The data supporting this article have been included as part of the supplementary information (SI). Supplementary information is available. See DOI: https://doi.org/10.1039/d5sc04715c.
