# Supplementary material for: Leveraging an intelligent slug flow platform for self-optimization of reaction systems with categorical variables
Source: Chem Sci. 2025 Oct 13;16(47):22447–55. doi: 10.1039/d5sc04715c (PMC12550597; doi:10.1039/d5sc04715c)
Supplement: SC-016-D5SC04715C-s001 [file SC-016-D5SC04715C-s001.pdf]

Supplementary Information (SI) for Chemical Science.
This journal is © The Royal Society of Chemistry 2025

Supporting Information

Leveraging an intelligent slug flow platform for self-optimization of reaction systems with categorical variables

Florian Wagner,[a,b] Gernot Neun,[a,b] Thomas Tampone,[c] Zhen Lei,[c] Frederic G. Buono,[c] Christopher A. Hone *[a,b] and C. Oliver Kappe*[a,b]

[a]*Center for Continuous Flow Synthesis and Processing (CCFLOW), Research Center Pharmaceutical Engineering GmbH (RCPE), Inffeldgasse 13, 8010 Graz, Austria. Email: christopher.hone@rcpe.at*

[b]*Institute of Chemistry, University of Graz, NAWI Graz, Heinrichstrasse 28, A-8010 Graz. Email: oliver.kappe@uni-graz.at*

[c]*Boehringer Ingelheim Pharmaceuticals, Inc, 900 Ridgebury Road, Ridgefield, CT 06877, USA.*

Table of Contents

1. General Experimental Details 3

1.1 Platform 3

2. Control Software 5

3. PAT Instruments 5

3.1 Online UHPLC 5

3.2 Inline FTIR 9

4. Setup 10

5. Simulation experiments 13

6. Self-Optimization Experiments 17

5.1 General Details 17

5.2 Reaction 1 (Amidation) 19

5.4 Reaction 2 (Haloform-type amidation) 22

7. Transfer to Continuous Flow 28

8. Batch Synthesis of Products 30

1. General Experimental Details

Solvents and chemicals were purchased from commercial suppliers and used without further purification except 2-Trichloroacetyl-1-methylpyrrole (purification protocol in batch section). Methyl cyanoacetate (purity >99.0%), 1,8-Diazabicyclo[5.4.0]-7-undecene (DBU, purity >98.0%) and triethylamine (TEA, >99.0%) were purchased from TCI. Piperidine (purity 99%) and 1,1,3,3-tetramethylguanidine (TMG, purity 99%), were purchased from Sigma-Aldrich. Pyridine (purity 99.5%) was purchased from Lab Scan. Dry pyridine (Pyr, purity 99.5+%) was purchased from ThermoFischer. 1-(o-Tolyl)biguanide (OTG, purity 97%) was purchased from abcr. 1,5,7-triazabicyclo[4.4.0]dec-5-ene (TBD, purity 97%), 5-Amino-2-fluorobenzonitrile (purity 98%), 1,5-Diazabicyclo[4.3.0]non-5-ene (DBN, purity 99.33%), 2-(tert-Butyl)-1,1,3,3-tetramethylguanidine (TbTMG, purity 98.74%) and 7-Methyl-1,5,7-triazabicyclo[4.4.0]dec-5-ene (Me-TBD, purity 95%) were purchased from BLDPharma. Methanol (purity 98.5%) and Dimethyl sulfoxide (DMSO, purity 99%) were purchased from VWR. 2-Methyltetrahydrofuran (Me-THF, purity 100%) was bought from Merck. Acetonitrile (HPLC grade) was bought from Sigma-Aldrich. Acetonitrile (dry MeCN, purity 99.9+%) was purchased from ThermoFischer.

1.1 Platform

The automation platform (Figure 1) is comprised of a Supervisory Control and Data Acquisition (SCADA) software (Evon, XAMControl), which is further connected to a Distributed Control System (DCS) (HiTec Zang, LabVision software and LabManager hardware), which communicates with actuators and sensors. The platform includes a syringe pump (SyrDos2 high-pressure pump head) and an HPLC pump (Knauer, AZURA P 4.1S with 10 mL pump heads made out of stainless steel, ceramic or Hastelloy). Reaction mixtures are prepared via a liquid handler (Gilson GX-241) and a syringe pump (Gilson Verity 4020 with 500 µL syringe). Additionally, the platform includes thermostats (Huber, Ministat 240), gas and liquid mass flow controllers (Bronkhorst) and pressure controllers (Bronkhorst, EL-PRESS). Several real-time PAT instruments such as pressure sensors, FTIR (Mettler Toledo, ReactIR) and online UHPLC (Shimadzu, Nexera X2) are established within the platform. Automated data processing is accomplished using PEAXACT and ProcessLink (S-PACT). Advanced Process Control is enabled by communication with Python scripts and XAMControl.

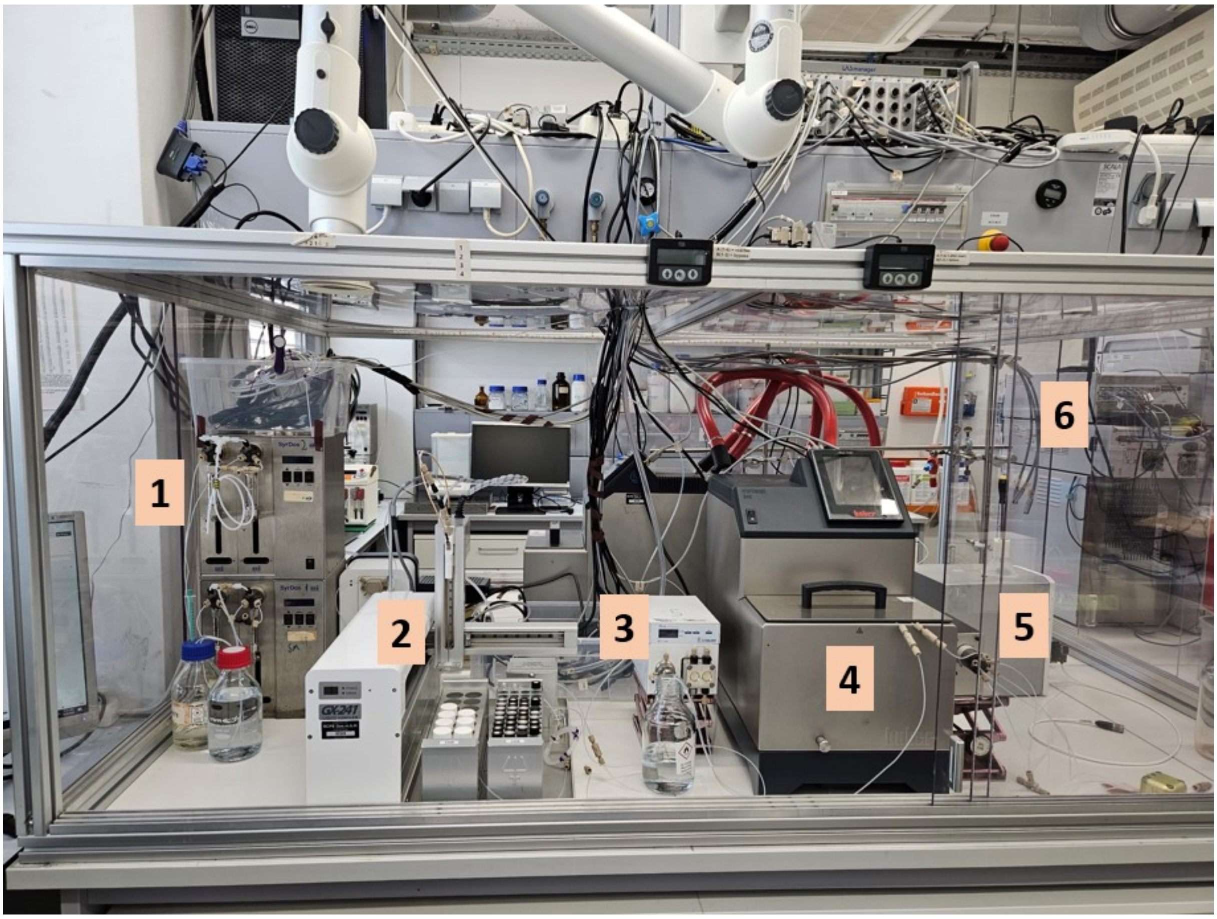

Figure 1 Photograph of the closed-loop self-optimization platform

Table 1 List of parts included in the platform

1	Syringe Pump SyrDos2 high-pressure pump head
2	Liquid Handler (Gilson GX-241) and Syringe Pump (Gilson Verity 4020 with 500 µL syringe)
3	HPLC Pump (Knauer, AZURA P 4.1S with 10 mL pump head)
4	Thermostat (Huber, Ministat 240)
5	FTIR (Mettler Toledo, ReactIR)
6	UHPLC (Shimadzu, Nexera X2)

2. Control Software

XAMControl is a SCADA software, allowing for direct communication with actuators and sensors utilizing different field bus protocols as well as OPC UA communication with various DCS systems. The process is visualized using XAMControl Iris, which allows for the display of real-time process data and manual process control. XAMControl allows for PLC integration using graphical programming and the C# programming language. All recorded data is stored in a cloud repository and can be accessed using XAMControl Iris or exported as .csv files.

3. PAT Instruments

3.1 Online UHPLC

The UHPLC (Shimadzu, Nexera X2) was comprised of a degassing unit (DGU-403ASR), two solvent delivery units (LC-30AD), a thermostated column oven (CTO-20AC), a diode array detector (SPD-M30A) and a control unit (CBM-20A). The analysis was carried out using a reversed-phase column (Phenomenex Luna Omega C18 (50 x 2.1 mm, particle size 1.6µm, pore size 100 Å)) at 45 °C using a total flow rate of 1 mL/min. The sample was introduced by an internal injection valve (10 nL, 20000 psi, Cheminert Nanovolume, Part# C84U-6674-.01EUH), which was triggered by the CBM-20A controller. Solvents used in UHPLC analysis were A: 10% MeCN in H2O + 0.1% formic acid and B: MeCN + 0.1% formic acid.

For the amidation reaction the following gradient was used:

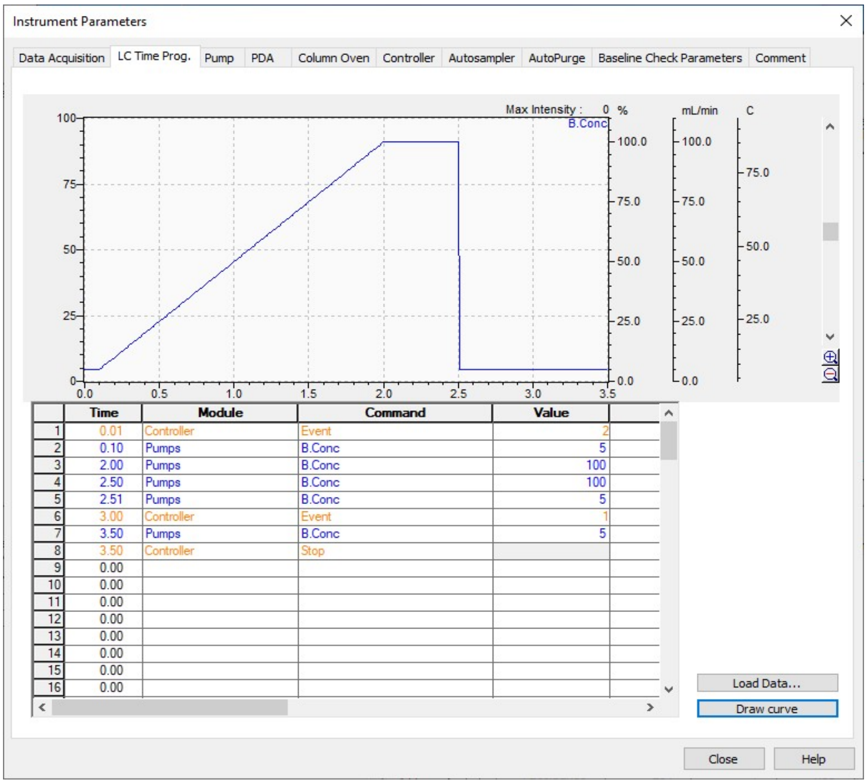

	Time	Module	Command	Value
1	0.01	Controller	Event	2
2	0.10	Pumps	B.Conc	5
3	2.00	Pumps	B.Conc	100
4	2.50	Pumps	B.Conc	100
5	2.51	Pumps	B.Conc	5
6	3.00	Controller	Event	1
7	3.50	Pumps	B.Conc	5
8	3.50	Controller	Stop	
9	0.00			
10	0.00			
11	0.00			
12	0.00			
13	0.00			
14	0.00			
15	0.00			
16	0.00			

Figure 2 Gradient of UHPLC method 1 used in the amidation reaction

For the haloform-type amidation the following gradient was used:

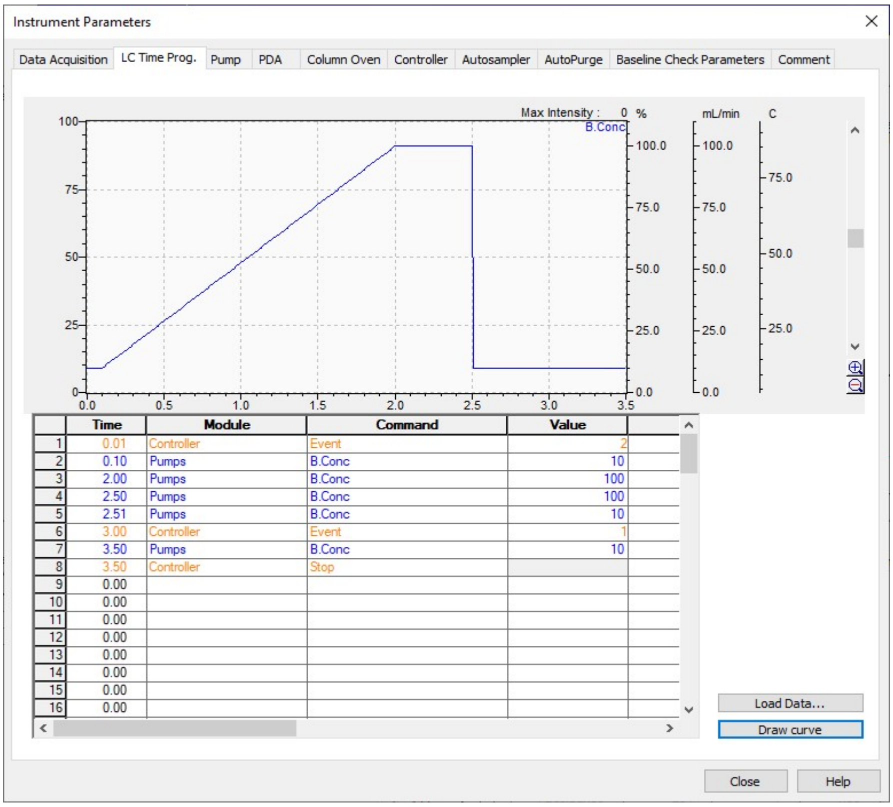

	Time	Module	Command	Value
1	0.01	Controller	Event	2
2	0.10	Pumps	B.Conc	10
3	2.00	Pumps	B.Conc	100
4	2.50	Pumps	B.Conc	100
5	2.51	Pumps	B.Conc	10
6	3.00	Controller	Event	1
7	3.50	Pumps	B.Conc	10
8	3.50	Controller	Stop	
9	0.00			
10	0.00			
11	0.00			
12	0.00			
13	0.00			
14	0.00			
15	0.00			
16	0.00			

Figure 3 Gradient of UHPLC method 3 for the haloform-type amidation

5-point calibration for products **3** and 3-point calibration for starting material **5** and product **6** were carried out.

Table 2 Concentrations of calibration solutions

Compounds	Calibration				
	1 (mol/L)	2 (mol/L)	3 (mol/L)	4 (mol/L)	5 (mol/L)
Product **3**	0.0025	0.025	0.125	0.25	0.3
Starting material **5**		0.025	0.125	0.25	
Product **6**	0.013	0.068	0.125		

Calibration curves are shown below in Figure 3, Figure 4 and Figure 5.

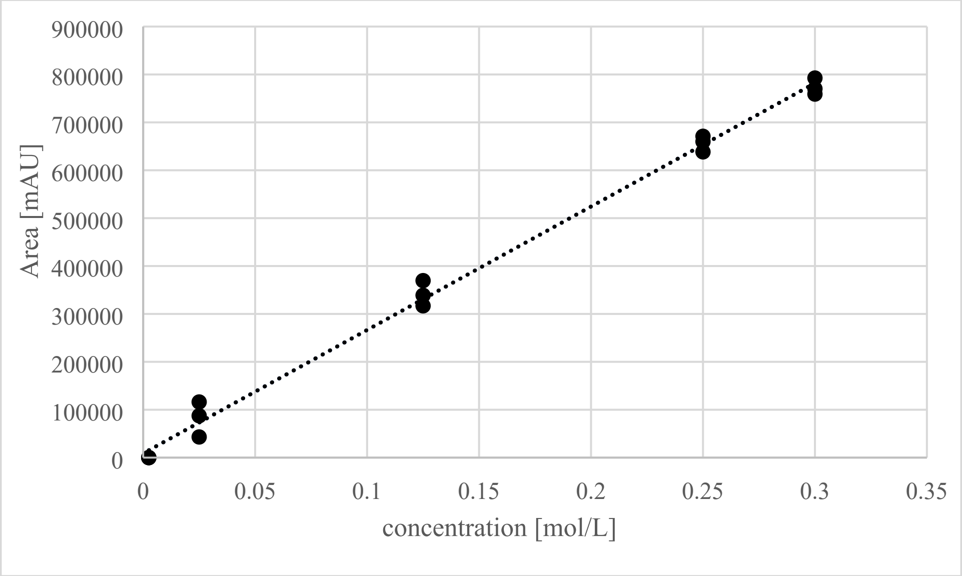

*Figure 3 Calibration curve for online UHPLC analysis of product **3***

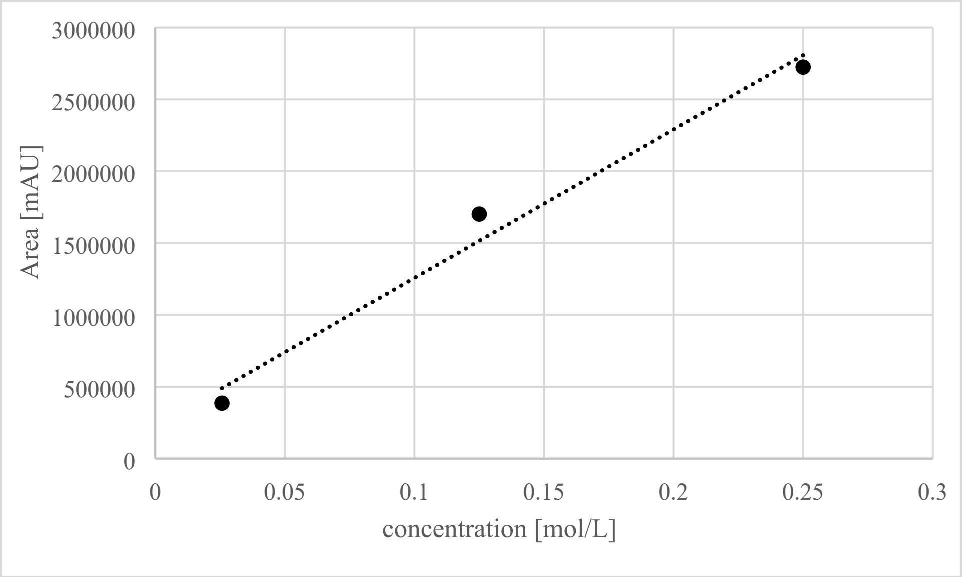

*Figure 4 Calibration curve for online UHPLC analysis of starting material **5***

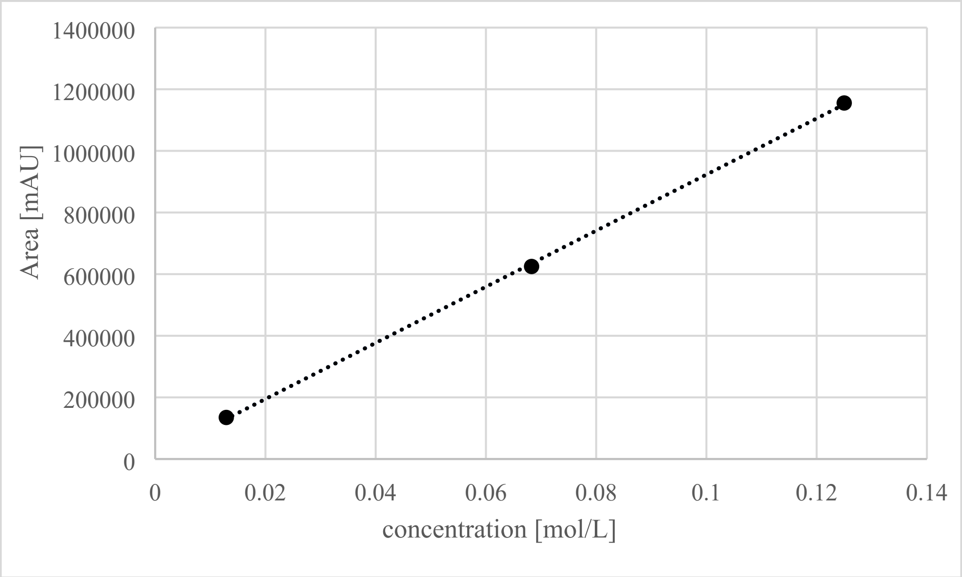

*Figure 5 Calibration curve of online UHPLC analysis of product **6***

UHPLC integration:

Online UHPLC integration was accomplished using a UHPLC internal sample injector (10 nL, 20000 psi, Cheminert Nanovolume, Part# C84U-6674-.01EUH), controlled using the Shimadzu LabSolutions Software. The chromatographic method engaged the injection valve upon receiving the start signal from the HiTec Zang LabVision software. This start signal was in turn triggered by a processed FTIR measurement. After each analysis the processed UHPLC data was automatically exported into a .csv file using the Shimadzu LabSolutions software (Version 5.97 SP1) containing information about retention times, areas of analytes and the chromatograms at 254 nm and 215 nm. This file was read by XAMControl and the data contained within was processed further.

3.2 Inline FTIR

General Details:

Inline FTIR Spectra were recorded using a Mettler Toledo ReactIR 702L FTIR (Figure 6), equipped with a flow cell (Mettler Toledo, DS Micro Flow Cell). The acquisition time was 5 sec per data point and the spectra were recorded between 600 cm^{-1} and 4000 cm^{-1} using a resolution of 4 cm^{-1}. It was ensured that the peak height was between 18000 and 24000 and that the signal to noise ratio was above 5000.

Process Integration:

The process stream was connected to the flow cell using a sampling system utilizing a remote-controlled VICI 6-port valve.

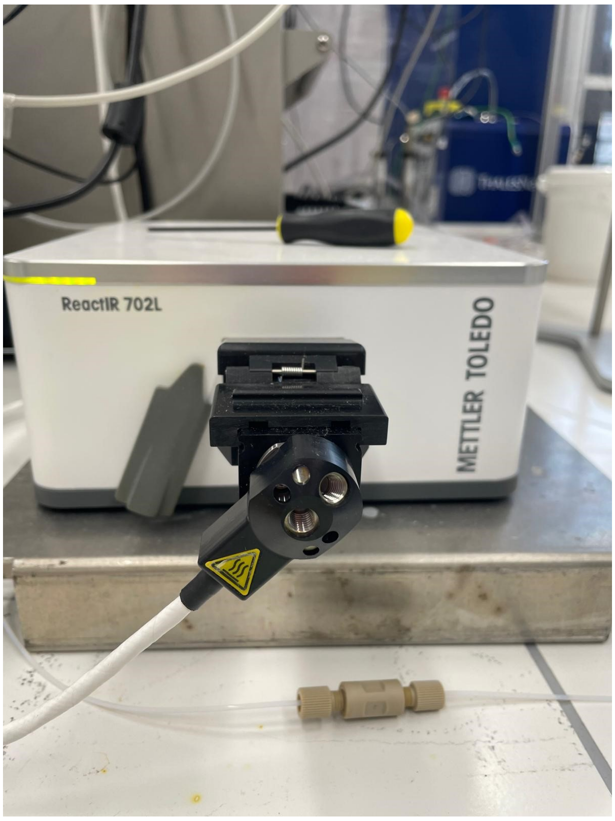

Figure 6 Photograph of the ReactIR 702L with flow cell

4. Setup

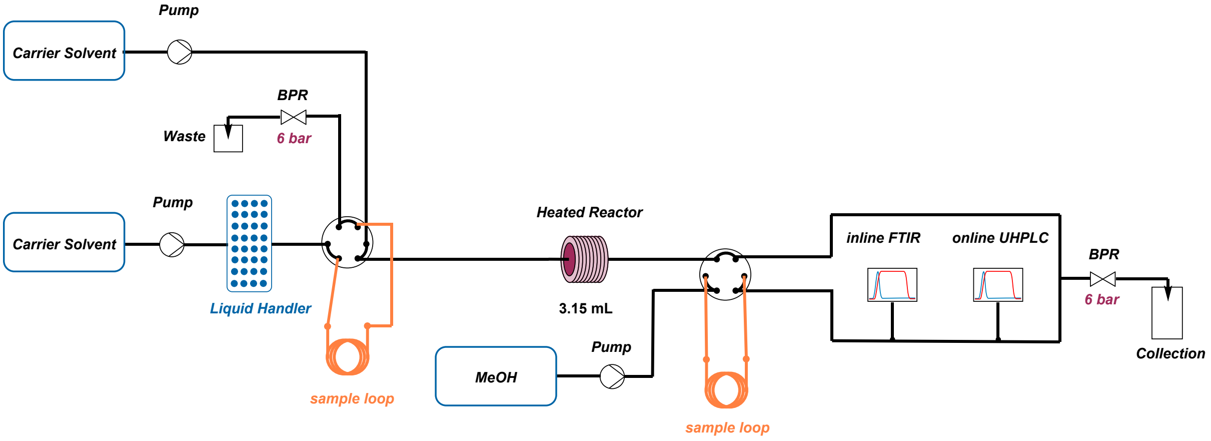

Figure 7 Detailed flow setup of the slug flow platform for self-optimization.

The self-optimization reactions were carried out in a coil reactor consisting of 1/16” PFA tubing, submerged in the oil bath of a Thermostat (Huber, Ministat240). The flow scheme is shown in Figure 7. Reaction mixtures where prepared in a reservoir (Figure 9) between the syringe pump (Gilson) and needle of the liquid handler. Following aspiration order was applied: N_2 gas, reaction mixture and N_2 gas again. The slug (gas-mixture-gas) entered the first 6-port valve and after passing through the valve, the reaction slug was positioned in a 3.5 mL sample loop of 1/16” PFA tubing before entering the thermostat. The slug was then pushed via an HPLC pump (Knauer) through the coil reactor. After passing through, the slug entered a second 6-port valve and into another sample loop. The reaction slug entered the loop and upon switching the valve was transferred to a separate flow system fed by a SyrDos2 syringe pump equipped with a pressure sensor (Keller, PAA 35XHTC). The reaction slug passed through the flow cell (Mettler Toledo, Micro Flow Cell DS SiComp) of the FTIR (Mettler Toledo, ReactIR 15), then the sample injector of the UHPLC (Shimadzu, Nexera X2). Upon exiting the injection valve, the reaction slug was transported to a membrane-based BPR (Zaiput, BPR-10, set to 6 bar) in 1/16” PFA tubing and collected after passing through the BPR.

4.2 FTIR Usage

The FTIR spectra were used to validate the shape and integrity of the reaction slug, as seen in Figure 8. Upon detecting steady state, the reaction mixture was injected into the UHPLC to analyze the reaction. For case study 1, IR traces were recorded at 1710 cm^{-1}, for case study 2 to IR traces were recorded at 980 cm^{-1}

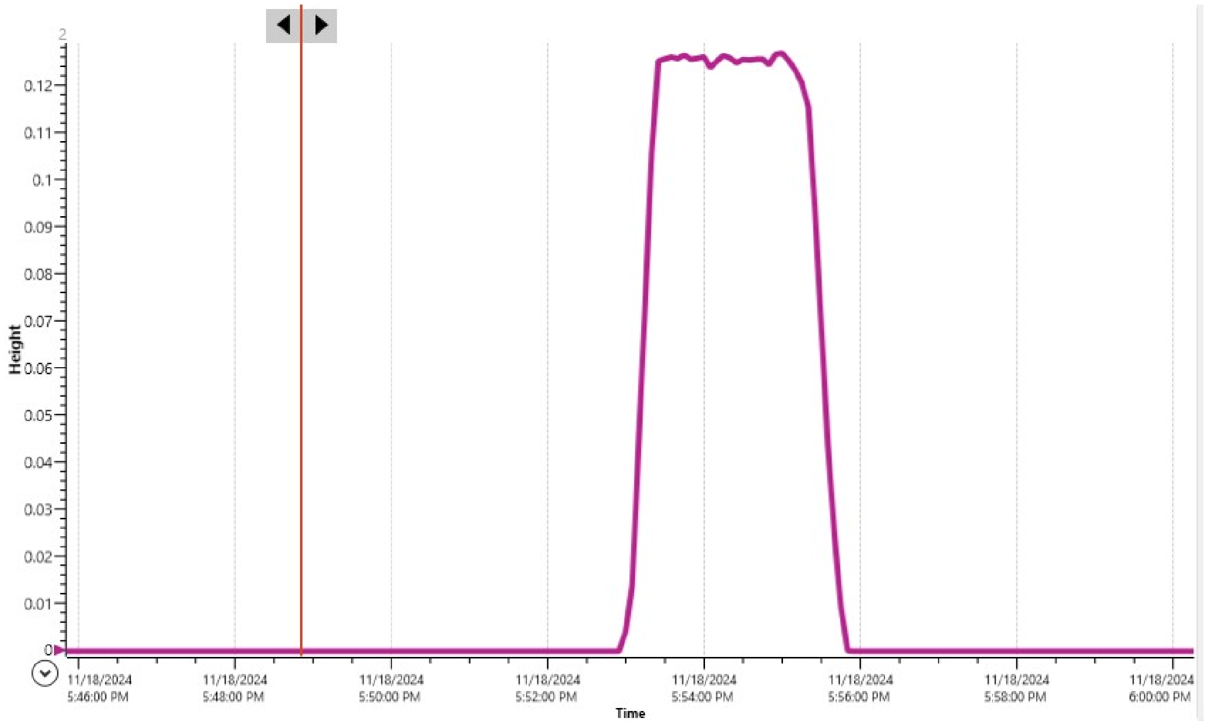

Figure 8 Example FTIR trace of individual iteration of case study 2 at 980 cm^{-1}

4.3 Liquid Handler Setup

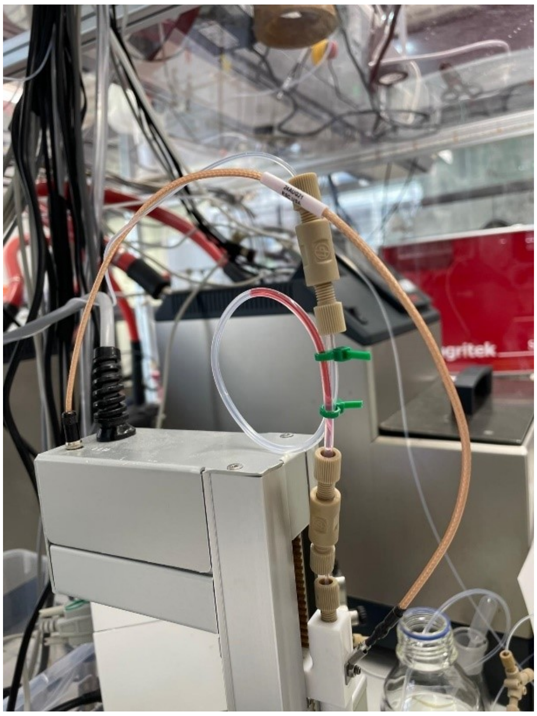

Figure 9 Reservoir for the preparation of reaction mixtures, eosin in MECN used as example.

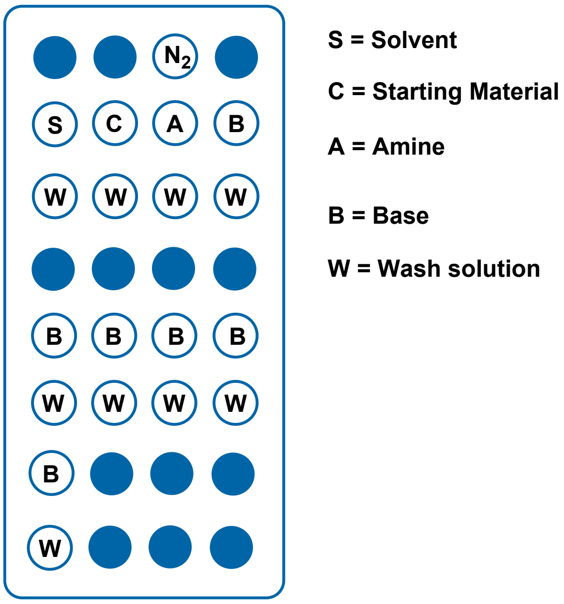

Figure 10 Detailed positions of stock solutions in the rack of the liquid handler.

In order to avoid cross-contamination and gas bubbles between aspirations, following method was developed to create reaction mixtures. After N_2 gas was aspirated from the inert gas vial into the reservoir to create a spacer for the front of the slug, the reaction mixture was made. First, half of the total amount of solvent was aspirated, followed by the educts, the base and then the second half of the solvent. Between each aspiration of liquids, the needle was inserted into the wash solution vials neighbouring the reactant stocks.

5. Simulation experiments

To verify the viability of this approach, a set of simulation studies was performed to compare different encoding approaches for a different number of categorical variables were investigated using a simulation strategy. For this simulation study a previously obtained kinetic model (Figure 11) was used in Bayesian optimization campaigns to compare the performance of different encoding methods, different lengths of the categorical vector and different optimization algorithms. All simulations were performed 20 times (except for the 9 simulated nucleophile simulation), initialized with the same Latin-hypercube sample each time, with the yield averaged. Simulations were performed using the TSEMO algorithm with default settings (exponential kernel, 1500 spectral samples, 10 retries) as provided in the Summit package[1] unless otherwise noted. Six different optimization variables were considered: Concentration of Ester starting material, equivalents of amine starting material, Concentration of nucleophilic base, reaction time and reaction temperature.

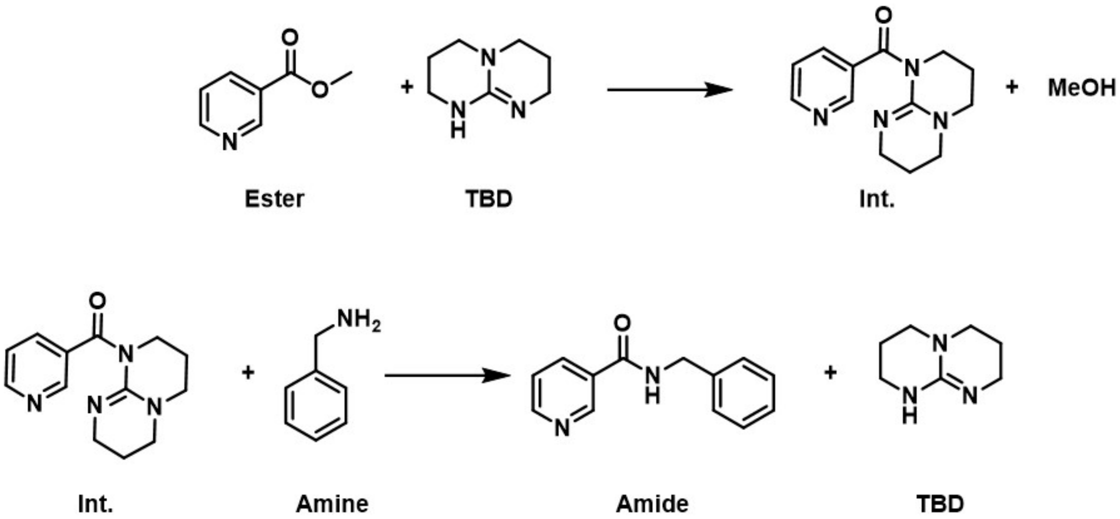

$$\frac{d[int]}{dt} = k_{Nu}\, \boldsymbol{A1} e^{\frac{-\boldsymbol{Ea1}}{RT}} [ester]^1\, [TBD]^1$$

$$\frac{d[product]}{dt} = \boldsymbol{A2} e^{\frac{-\boldsymbol{Ea2}}{RT}} [amine]^1\, [int]^1$$

Fitted parameters:
A1 = 1.747191
A2 = 0.164510
Ea1 = 17.698
Ea2 = 0.169

Figure 11 Kinetic model[2] used in simulation experiments. Kinetic parameters used are based on a previous publication. k_{Nu} represents a nucleophilicity-derived parameter to simulate the impact of different nucleophile strengths on the reaction. k_{Nu} values were derived based on values obtained from the Nucleophilicity database[3]

According to the Mayr-Patz Equation:

$$\log k = s(N + E)$$

where k is the rate constant, s is an electrophile-derived slope factor and N and E are nucleophilicity and electrophilicity of the reactants), the reaction rate is correlated with the nucleophilicity and electrophilicity of the reactants. If the electrophile reactant is unchanged and only the relative Nucleophilicity of two different reactants is considered this equation simplifies to:

$$\frac{k}{k_0} = 10^{N - N_0} = k_{Nu}$$

The model used in the simulation campaigns was acquired using TBD as nucleophilic catalyst, meaning $k_{TBD} = k_0$.

Table 3 Nucleophilicity and kNu values used in simulated optimization campaigns

Base	N	kNu*
TBD	16.2	1
1,2,3,5,6,7-hexahydroimidazo[1,2-a]pyrimidine	16.15	0.97
2,3,5,6-tetrahydro-1H-imidazo[1,2-a]imidazole	14.44	0.02
2-benzyl-1,1,3,3-tetramethylguanidine	14.36	0.016
N-(1,3-dimethylimidazolidin-2-ylidene)-1-phenylmethanamine	14	0.007
1,1,3,3-tetramethylguanidine	13.58	0.003

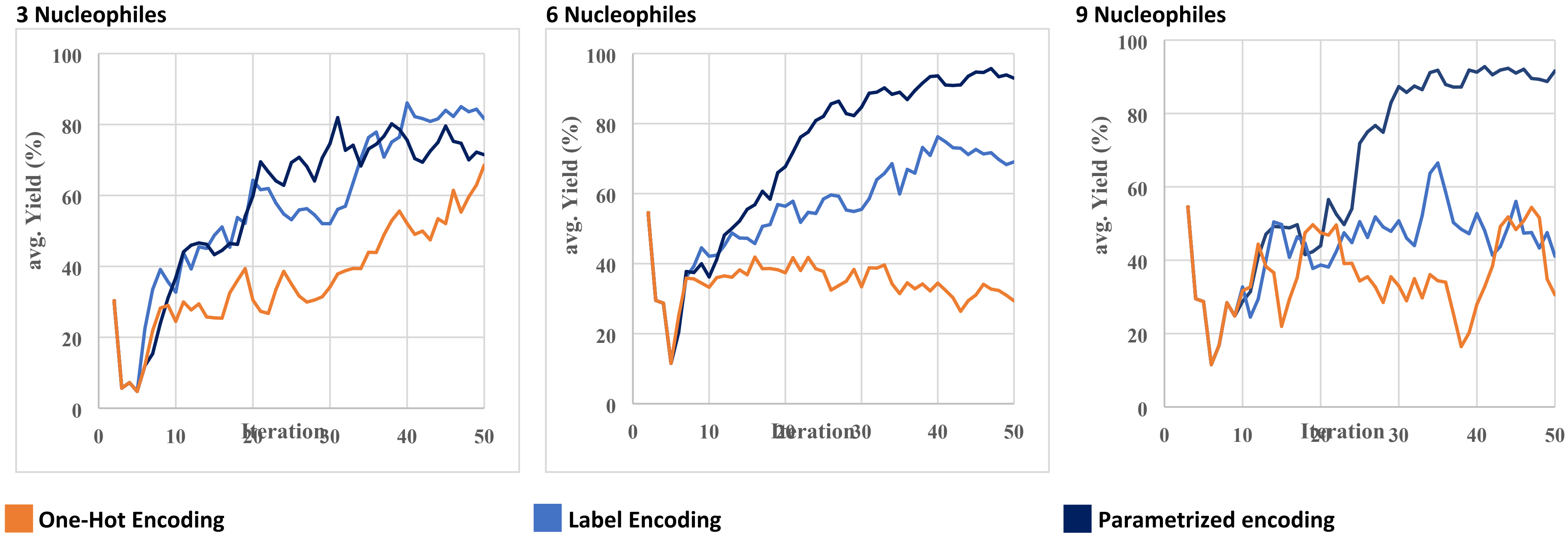

Figure 12 Comparison of different encoding methods for different sizes of the categorical vector

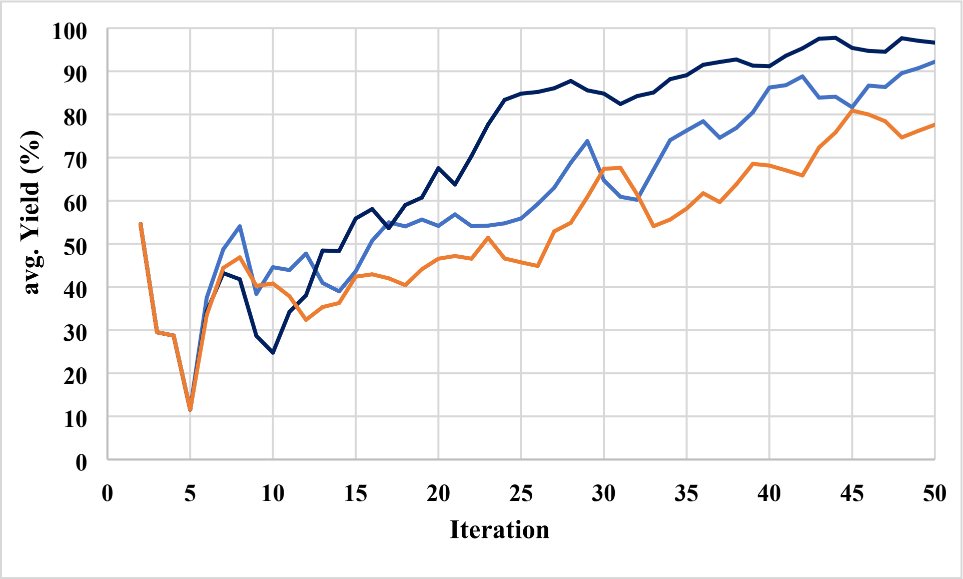

Figure 13 Comparison of different encoding methods using expected improvement as the acquisition function in Bayesian optimization, with a categorical vector length of 6

As shown in Figure 12 and Figure 13 in the simulation the parametrized approach reliably outperforms one-hot encoding. The parametrized approach also performs better than Label encoding at longer lengths of the categorical vector. As expected, the performance of the expected improvement-based SOBO algorithm is similar (Figure 13), but the parametrized encoding method still improves more quickly initially and reaches a plateau faster.

6. Self-Optimization Experiments

5.1 General Details

During the self-optimization experiments the optimization algorithm could adjust 6 different variables: RT (residence time in reactor), T (temperature of the reactor), Conc SM (concentration of starting material), R1/SM (ratio of amine to starting material), Base Type (base to choose from the set), Ratio Base (ratio of base to starting material). The boundaries of the adjustable variables were varied based on the reaction and experiment and are shown below. The boundaries of the continuous variables were determined based on chemical and analytical limitations (concentration and equivalents) and platform limitation (temperature and reaction times).

In all experimental case studies, the TSEMO algorithm as implemented in Summit was used with default settings (exponential kernel, 1500 spectral samples, 10 retries). Continuous variables were passed directly to the optimization algorithm without further transformation. The discrete numerical values denoting the base type in both the label encoding and the chemical descriptor-based encoding were first log transformed the normalized to obtain optimization variable values between 0 and 1. Discrete numerical variables were selected based on the smallest 1-dimensional distance from the algorithm suggestion.

In all case studies, the output of the reaction was normalized prior to passing it to the algorithm.

Algorithm implementation:

```
import summit.domain as domain
class constraints:
    ratioAmine = domain.ContinuousVariable("Ratio_Amine", "Ratio of Amine to acid SM", [0.5, 1.5])
    conc_SM = domain.ContinuousVariable("Conc_SM", "concentration of acid SM", [0.1, 0.30])
    tres = domain.ContinuousVariable("Residence_Time", "Residence Time", [0.5, 5])
    temperature = domain.ContinuousVariable("Temperature", "Temperature", [10, 200])
    ratioCat = domain.ContinuousVariable("ratio_cat", "Ratio of catalyst to ester SM", [0.05, 0.5])
    nucleophilicity = domain.ContinuousVariable("Nucleophilicity", "Nucleophilicity of reagent", [-6, 0])
    yld = domain.ContinuousVariable("Yield", "yield", [0, 1], is_objective = True)

    vars = [ratioAmine, conc_SM, tres, temperature, ratioCat, nucleophilicity, yld]
    dom = domain.Domain(vars)

    def getDomain(self):
        return self.dom

    def getCols(self):
        domdict = self.dom.to_dict()
        Names = []

        for i in range(len(domdict)):
            Names.append(domdict[i].get('name'))
        return Names
```

Code Snippet 1 Implementation of the optimization constraints class

```python
from summit.strategies import TSEMO
import constraints as constraints

class TSEMO_iterator:
    def __init__(self):
        self.con = constraints.constraints()
        self.strategy = TSEMO(self.con.getDomain())

    def suggest_next(self, previous):
        next_experiment = self.strategy.suggest_experiments(1,prev_res=previous)
        return next_experiment
```

Code Snippet 2 Implementation of the experiment suggestion class using Summit

5.2 Reaction 1 (Amidation)

The adjustable variables had the following boundaries (Table 4).

*Table 4 Lower and upper boundaries in the self-optimization experiments for Reaction **1***

Limits	RT	T	Conc SM	Ratio R1/SM	Ratio Base	Base Type
	(min)	(°C)	(mol/L)			
Upper	12	100	0.25	2.0	1.2	6 options
Lower	2	20	0.1	1.0	0.2	

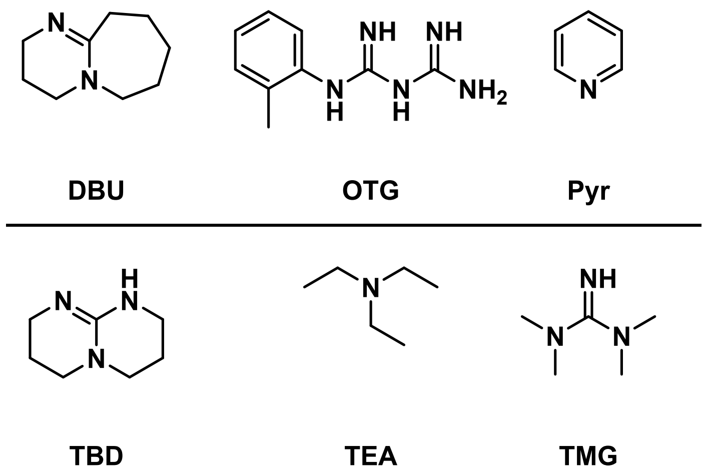

*Figure 14 Catalysts for Reaction **1***

Table 5 Replicates of initialization experiment 4 for Reaction 1

Replicate Index	RT (min)	T (°C)	Conc SM (mol/L)	Ratio R1/SM	Ratio Base	Base Type	Yield (%)
1	5.26	69.36	0.22	1.6	1.05	TBD	31.64
2	5.26	69.36	0.22	1.6	1.05	TBD	32.26
3	5.26	69.36	0.22	1.6	1.05	TBD	29.17

*Table 6 Self-optimization using the TSEMO algorithm for Reaction **1** and Latin hypercube as initial data set. Bases label-encoded. Highest yield highlighted in yellow.*

Expt. Type	Expt #	RT (min)	T (°C)	Conc SM (mol/L)	Ratio R1/SM	Base Type	Ratio Base	Yield (%)
LHC	1	8.88	23.16	0.12	1.50	TMG	1.03	10.97
LHC	2	2.97	33.52	0.16	2.00	TBD	0.23	7.62
LHC	3	7.51	55.95	0.18	1.83	Pyr	0.63	0.27
LHC	4	5.26	69.36	0.22	1.60	TBD	1.05	31.64
LHC	5	4.48	77.61	0.11	1.32	Pyr	0.78	12.57
LHC	6	8.45	99.42	0.20	1.37	TMG	0.60	16.27
LHC	7	5.38	27.34	0.24	1.90	TEA	0.46	15.82
LHC	8	6.39	42.68	0.13	1.08	OTG	0.81	0.00
LHC	9	11.16	51.31	0.17	1.50	TEA	0.39	5.72
LHC	10	11.40	66.13	0.15	1.12	DBU	0.93	9.98
LHC	11	9.51	80.73	0.20	1.20	DBU	1.12	19.52
LHC	12	2.50	87.11	0.23	1.71	OTG	0.36	7.22
TSEMO	13	6.70	37.01	0.17	1.68	TMG	0.29	0.33
TSEMO	14	10.65	70.23	0.23	1.71	TEA	0.92	35.31
TSEMO	15	6.62	74.08	0.23	1.63	TMG	0.55	19.27
TSEMO	16	4.32	55.01	0.23	1.61	TMG	1.18	15.20
TSEMO	17	8.21	84.45	0.21	1.77	TEA	0.86	30.03
TSEMO	18	4.97	100.00	0.17	1.64	TEA	0.49	6.36
TSEMO	19	2.15	91.46	0.22	1.52	OTG	0.89	10.95
TSEMO	20	6.78	63.43	0.23	2.00	TEA	0.46	26.18
TSEMO	21	4.61	69.07	0.23	1.96	OTG	0.69	13.63
TSEMO	22	8.38	63.35	0.23	1.73	TBD	0.92	30.98

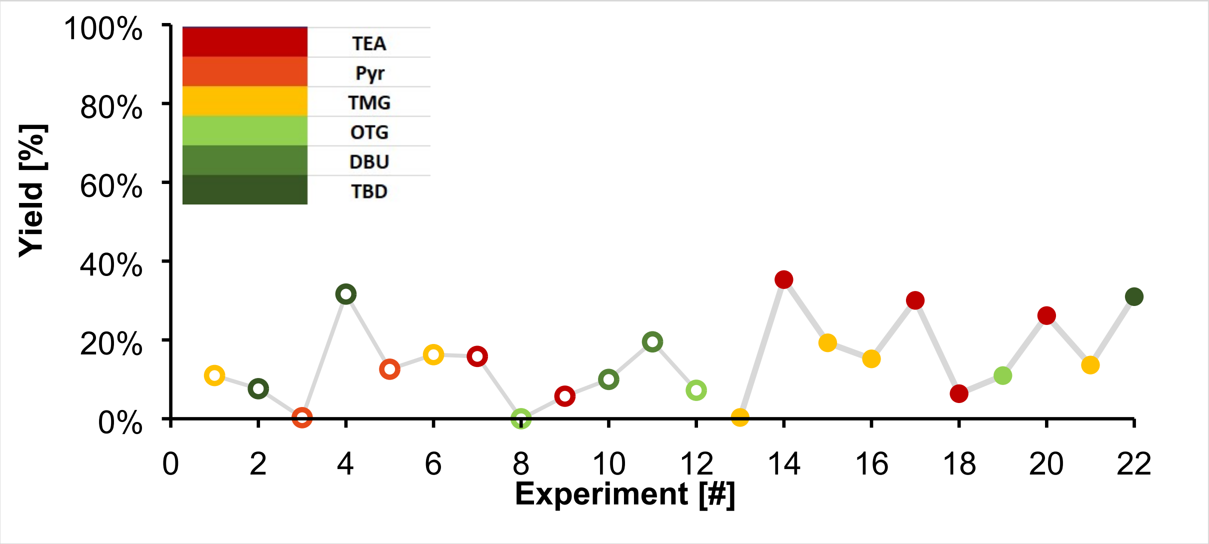

*Figure 15 Results of Reaction **1** with label-encoded catalysts, unfilled circles denote LHC initialization*

*Table 7 Self-optimization using the TSEMO algorithm for Reaction **1** and Latin hypercube as initial data set. Bases semi continuously encoded. Highest yield highlighted in yellow.*

Expt. Type	Expt #	RT (min)	T (°C)	Conc SM (mol/L)	Ratio R1/SM	Base Type	Ratio Base	Yield (%)
LHC	1	8.88	23.16	0.12	1.50	TMG	1.03	10.97
LHC	2	2.97	33.52	0.16	2.00	TBD	0.23	7.62
LHC	3	7.51	55.95	0.18	1.83	Pyr	0.63	0.27
LHC	4	5.26	69.36	0.22	1.60	TBD	1.05	31.64
LHC	5	4.48	77.61	0.11	1.32	Pyr	0.78	12.57
LHC	6	8.45	99.42	0.20	1.37	TMG	0.60	16.27
LHC	7	5.38	27.34	0.24	1.90	TEA	0.46	15.82
LHC	8	6.39	42.68	0.13	1.08	OTG	0.81	0.00
LHC	9	11.16	51.31	0.17	1.50	TEA	0.39	5.72
LHC	10	11.40	66.13	0.15	1.12	DBU	0.93	9.98
LHC	11	9.51	80.73	0.20	1.20	DBU	1.12	19.52
LHC	12	2.50	87.11	0.23	1.71	OTG	0.36	7.22
TSEMO	13	4.50	94.71	0.24	1.74	TBD	0.81	45.80
TSEMO	14	4.14	74.22	0.18	1.83	DBU	0.99	20.05
TSEMO	15	3.43	70.81	0.22	2.00	TBD	0.91	35.88
TSEMO	16	6.12	84.41	0.24	1.85	TBD	0.85	30.12
TSEMO	17	3.20	74.88	0.12	1.37	OTG	1.04	9.15
TSEMO	18	8.10	82.10	0.12	1.05	Pyr	0.69	10.78
TSEMO	19	3.90	76.34	0.23	1.80	TBD	0.74	42.80
TSEMO	20	4.45	94.05	0.24	1.82	TBD	0.81	45.47
TSEMO	21	11.31	59.38	0.22	1.07	OTG	0.24	4.37
TSEMO	22	4.64	100.00	0.24	1.34	TBD	0.75	36.03

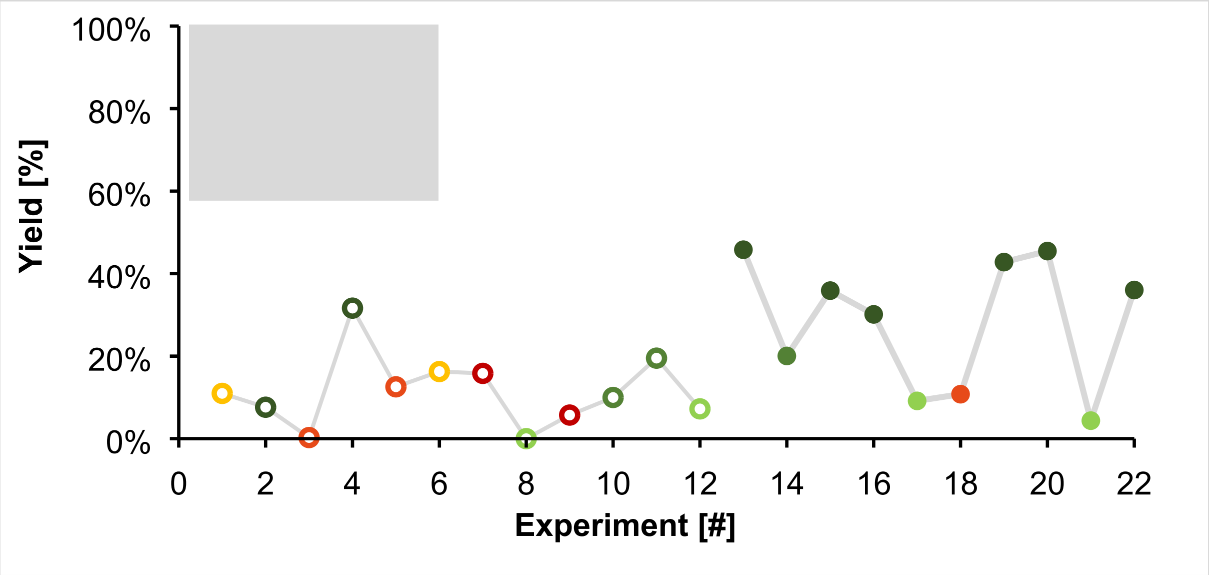

*Figure 16 Results of Reaction **1** with semi-continuous encoded catalysts, unfilled circles denote LHC initialization*

5.4 Reaction 2 (Haloform-type amidation)

Next to the single-objective optimization (yield) a multi-objective optimization campaign was done, simultaneously maximizing yield, while minimizing process mass intensity (PMI/mL) and the cost of base per mass of product (Cost Base/gP). Prices of the respective bases were obtained from Sigma-Aldrich (accessed December 2024) at comparable container sizes:

Table 8 Prices of bases used in multi-objective self-optimization

Base	Price per g [€]
TBD	14.8
DBU	0.7
MeTBD	74.3
DBN	1.43
TbTMG	29.0
Pyr	0.7

The adjustable variables had the following boundaries (Table 9).

*Table 9 Lower and upper boundaries in the self-optimization experiments for Reaction **2***

Limits	RT	T	Conc SM	Ratio R1/SM	Ratio Base	Base Type
	(min)	(°C)	(mol/L)			
Upper	14	120	0.2	1.5	2.5	6 options
Lower	2	30	0.1	0.5	0.2	

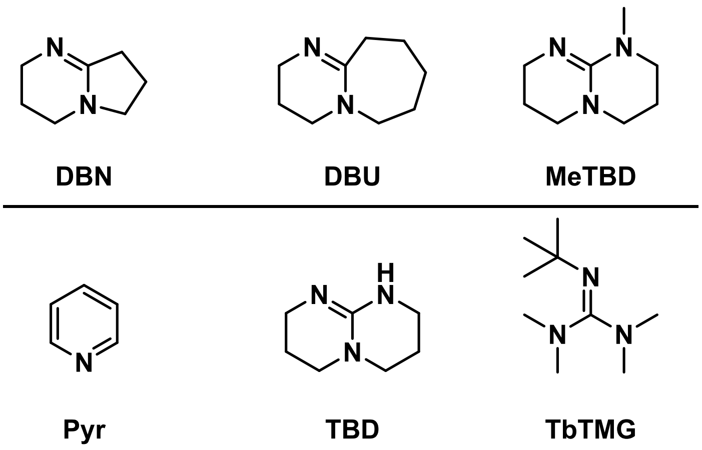

*Figure 17 Catalysts for Reaction **2***

Table 10 Replicates of initialization experiment 1 for Reaction 2

Replicate Index	RT (min)	T (°C)	Conc SM (mol/L)	Ratio R1/SM	Ratio Base	Base Type	Yield (%)
1	4.46	33	0.23	0.71	1.13	TBD	48.18
2	4.46	33	0.23	0.71	1.13	TBD	44.98
3	4.46	33	0.23	0.71	1.13	TBD	49.91

*Table 11 Single-objective self-optimization using the TSEMO algorithm for Reaction **2** and Latin hypercube as initial data set. Bases label-encoded. Highest yield highlighted in yellow.*

Expt. Type	Expt #	RT	T	Conc SM	Ratio R1/SM	Base Type	Ratio Base	Yield
		(min)	(°C)	(mol/L)				(%)
LHC	1	4.46	33	0.23	0.71	TBD	1.13	48.18
LHC	2	2.08	43	0.20	0.60	Pyr	2.09	0.00
LHC	3	8.64	59	0.15	0.68	DBU	0.75	3.71
LHC	4	7.32	90	0.21	1.01	TBD	2.44	41.53
LHC	5	12.36	99	0.21	1.13	Pyr	1.88	0.00
LHC	6	5.49	91	0.16	0.83	TbTMG	1.56	15.50
LHC	7	13.26	81	0.13	0.90	MeTBD	0.97	5.09
LHC	8	9.75	72	0.17	1.31	DBN	2.29	10.79
LHC	9	3.09	51	0.19	0.53	DBN	0.37	0.00
LHC	10	6.10	41	0.12	1.38	TbTMG	1.37	9.29
LHC	11	10.38	65	0.17	1.05	MeTBD	1.32	5.45
LHC	12	11.81	104	0.14	1.18	DBU	0.49	5.60
TSEMO	13	5.21	55	0.20	0.47	TbTMG	1.45	19.41
TSEMO	14	8.98	110	0.23	1.10	MeTBD	1.64	15.41
TSEMO	15	13.44	66	0.16	0.85	Pyr	2.01	0.00
TSEMO	16	11.99	96	0.21	1.12	Pyr	0.36	0.00
TSEMO	17	11.20	57	0.12	0.93	DBU	0.20	0.08
TSEMO	18	7.21	101	0.18	1.04	TbTMG	2.14	32.09
TSEMO	19	7.71	45	0.21	0.89	DBN	2.41	7.00
TSEMO	20	6.38	83	0.23	1.26	TbTMG	1.86	28.97
TSEMO	21	8.96	42	0.14	0.72	TBD	0.66	39.84
TSEMO	22	3.96	46	0.21	0.98	DBU	0.71	4.69
TSEMO	23	7.34	79	0.11	0.85	Pyr	0.61	0.00
TSEMO	24	2.66	105	0.18	0.66	DBN	2.33	12.56
TSEMO	25	10.33	103	0.21	0.90	TbTMG	1.78	0.16
TSEMO	26	7.86	93	0.20	0.93	TbTMG	1.83	32.93

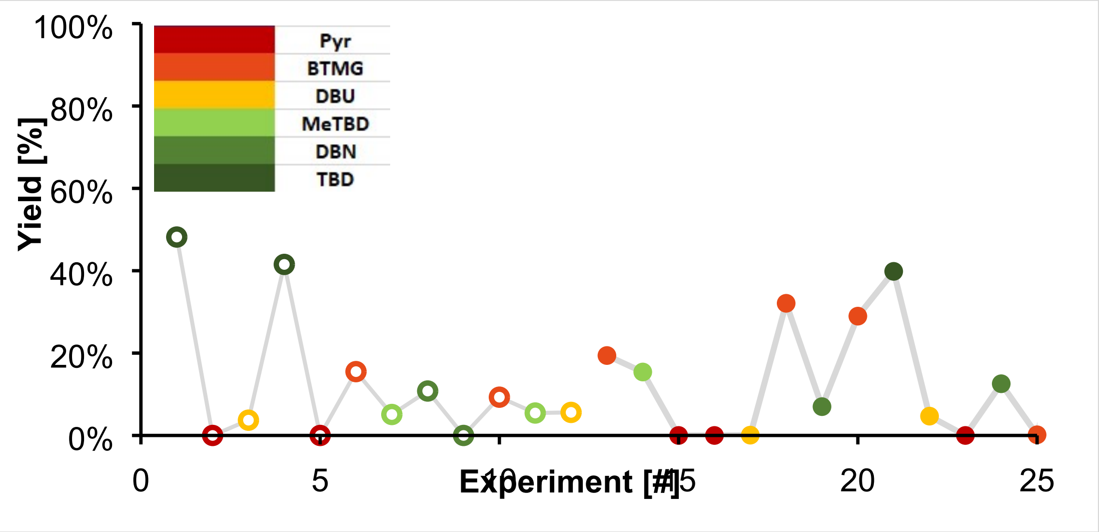

*Figure 18 Results of Reaction **2** with label-encoded catalysts, unfilled circles denote LHC initialization*

*Table 12 Single-objective self-optimization using the TSEMO algorithm for Reaction **2** and Latin hypercube as initial data set. Bases semi continuously encoded. Highest yield highlighted in yellow.*

Expt. Type	Expt #	RT (min)	T (°C)	Conc SM (mol/L)	Ratio R1/SM	Base Type	Ratio Base	Yield (%)
LHC	1	4.46	33	0.23	0.71	TBD	1.13	48.18
LHC	2	2.08	43	0.20	0.60	Pyr	2.09	0.00
LHC	3	8.64	59	0.15	0.68	DBU	0.75	3.71
LHC	4	7.32	90	0.21	1.01	TBD	2.44	41.53
LHC	5	12.36	99	0.21	1.13	Pyr	1.88	0.00
LHC	6	5.49	91	0.16	0.83	TbTMG	1.56	15.50
LHC	7	13.26	81	0.13	0.90	MeTBD	0.97	5.09
LHC	8	9.75	72	0.17	1.31	DBN	2.29	10.79
LHC	9	3.09	51	0.19	0.53	DBN	0.37	0.00
LHC	10	6.10	41	0.12	1.38	TbTMG	1.37	9.29
LHC	11	10.38	65	0.17	1.05	MeTBD	1.32	5.45
LHC	12	11.81	104	0.14	1.18	DBU	0.49	5.60
TSEMO	13	6.91	82	0.17	1.07	TBD	2.07	48.25
TSEMO	14	11.77	49	0.20	1.18	TBD	0.61	53.18
TSEMO	15	3.66	83	0.10	1.15	Pyr	0.75	0.00
TSEMO	16	10.81	62	0.20	0.80	TBD	1.33	66.34
TSEMO	17	7.68	107	0.20	1.21	DBN	1.16	22.49
TSEMO	18	9.59	47	0.20	0.66	MeTBD	0.56	3.99
TSEMO	19	10.24	67	0.20	1.10	TBD	1.78	52.25
TSEMO	20	12.23	64	0.20	0.81	TBD	0.89	63.41
TSEMO	21	13.10	70	0.17	0.82	TBD	1.25	59.23
TSEMO	22	9.14	75	0.19	0.69	TBD	1.10	67.19

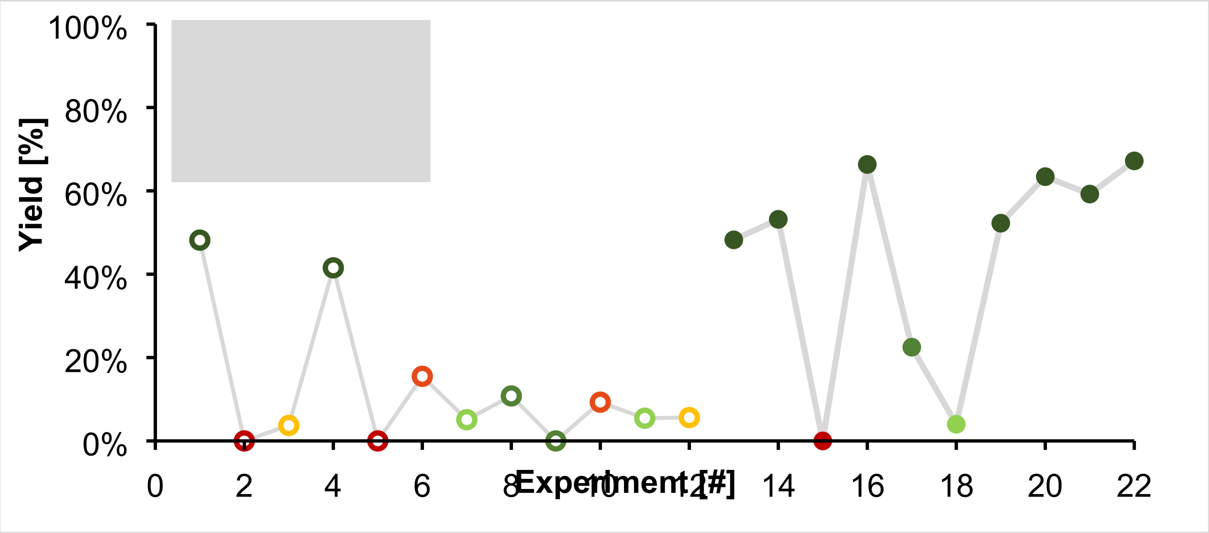

Figure 19 Results of Reaction 3 with semi-continuous encoded catalysts, unfilled circles denote LHC initialization

*Table 13 Multi-objective self-optimization using the TSEMO algorithm for Reaction **2** and Latin hypercube as initial data set. Bases one-hot encoded. Highest yield highlighted in yellow.*

Expt. Type	Expt #	RT	T	Conc SM	Ratio R1/SM	Base Type	Ratio Base	Yield	PMI/mL	Cost Base/g P
		(min)	(°C)	(mol/L)				(%)	(1/mL)	(€/g)
LHC	1	4.46	33.09	0.20	0.76	TBD	1.13	48.18	0.05	0.12
LHC	2	2.08	43.49	0.17	0.65	Pyr	2.09	0.00	1.00	0.50
LHC	3	8.64	59.11	0.13	0.73	DBU	0.75	3.71	1.00	0.50
LHC	4	7.32	89.90	0.18	1.08	TBD	2.44	41.53	0.05	0.23
LHC	5	12.36	99.48	0.18	1.21	Pyr	1.88	0.00	1.00	0.50
LHC	6	5.49	90.87	0.14	0.89	TbTMG	1.56	15.50	0.19	0.27
LHC	7	13.26	80.57	0.12	0.96	MeTBD	0.97	5.09	0.61	0.17
LHC	8	9.75	72.14	0.15	1.41	DBN	2.29	10.79	0.22	0.50
LHC	9	3.09	50.67	0.17	0.57	DBN	0.37	0.00	1.00	0.00
LHC	10	6.10	40.89	0.11	1.48	TbTMG	1.37	9.29	0.36	0.36
LHC	11	10.38	64.89	0.15	1.12	MeTBD	1.32	5.45	0.45	0.21
LHC	12	11.81	104.35	0.12	1.27	DBU	0.49	5.60	0.51	0.50
TSEMO	13	8.70	57.62	0.20	1.06	DBU	1.17	11.00	0.17	0.500
TSEMO	14	3.45	100.37	0.11	1.08	Pyr	2.50	0.00	1.00	0.500
TSEMO	15	2.00	55.76	0.18	1.46	Pyr	1.74	0.00	1.00	0.500
TSEMO	16	6.05	101.33	0.19	0.78	TbTMG	2.18	38.75	0.06	0.157
TSEMO	17	12.93	72.24	0.18	0.96	DBU	0.82	12.59	0.16	0.285
TSEMO	18	4.71	102.49	0.18	1.18	DBU	2.44	21.86	0.10	0.500
TSEMO	19	7.86	37.07	0.17	0.69	DBU	1.45	7.88	0.39	0.500
TSEMO	20	5.56	61.04	0.18	1.05	TBD	0.20	19.80	0.10	0.048
TSEMO	21	4.99	74.43	0.16	0.67	DBU	1.89	11.46	0.29	0.500
TSEMO	22	3.27	48.85	0.18	1.19	TbTMG	1.59	15.42	0.14	0.436
TSEMO	23	5.19	59.14	0.19	1.00	MeTBD	1.67	7.76	0.24	0.500
TSEMO	24	5.95	101.38	0.18	1.01	DBU	1.61	19.35	0.10	0.285
TSEMO	25	7.51	82.22	0.19	1.23	TbTMG	2.41	33.03	0.06	0.345
TSEMO	26	9.29	37.99	0.20	1.14	TbTMG	0.64	14.06	0.13	0.215
TSEMO	27	7.57	95.09	0.15	0.88	TBD	2.45	44.93	0.06	0.239

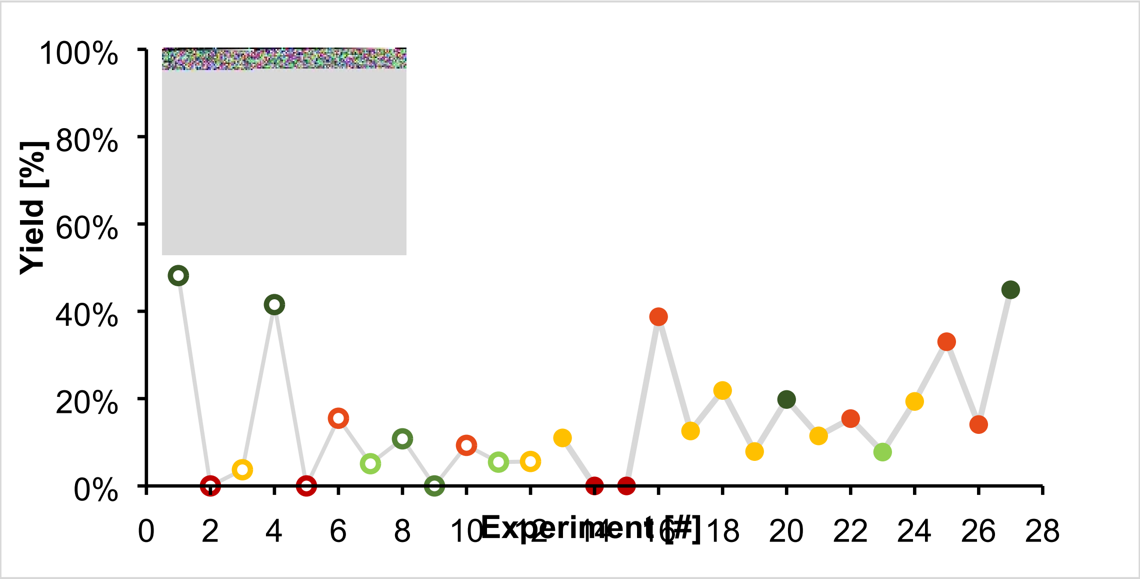

*Figure 20 Multi-objective results of Reaction **2** with label-encoded catalysts, unfilled circles denote LHC initialization*

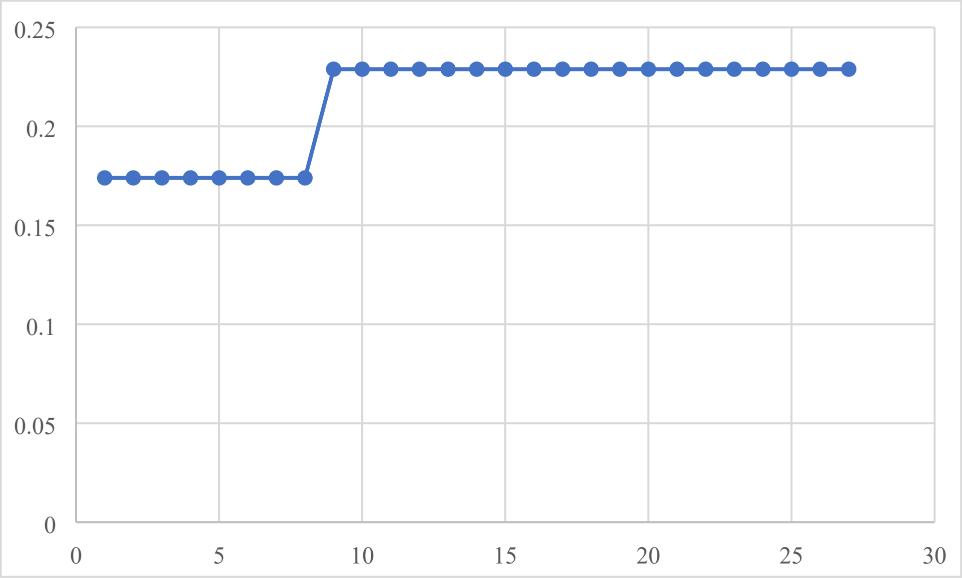

Figure 21 Improvement of hypervolume for reaction 2 with label encoded catalysts

*Table 14 Multi-objective self-optimization using the TSEMO algorithm for Reaction **2** and Latin hypercube as initial data set. Bases semi continuously encoded. Highest yield highlighted in yellow.*

Expt. Type	Expt #	RT	T	Conc SM	Ratio R1/SM	Base Type	Ratio Base	Yield	PMI/mL	Cost Base/g P
		(min)	(°C)	(mol/L)				(%)	(1/mL)	(€/g)
LHC	1	4.46	33.09	0.20	0.76	TBD	1.13	48.18	0.05	0.12
LHC	2	2.08	43.49	0.17	0.65	Pyr	2.09	0.00	1.00	0.50
LHC	3	8.64	59.11	0.13	0.73	DBU	0.75	3.71	1.00	0.50
LHC	4	7.32	89.90	0.18	1.08	TBD	2.44	41.53	0.04	0.23
LHC	5	12.36	99.48	0.18	1.21	Pyr	1.88	0.00	1.00	0.50
LHC	6	5.49	90.87	0.14	0.89	TbTMG	1.56	15.50	0.18	0.27
LHC	7	13.26	80.57	0.12	0.96	MeTBD	0.97	5.09	0.57	0.17
LHC	8	9.75	72.14	0.15	1.41	DBN	2.29	10.79	0.19	0.50
LHC	9	3.09	50.67	0.17	0.57	DBN	0.37	0.00	1.00	0.50
LHC	10	6.10	40.89	0.11	1.48	TbTMG	1.37	9.29	0.31	0.36
LHC	11	10.38	64.89	0.15	1.12	MeTBD	1.32	5.45	0.39	0.21
LHC	12	11.81	104.35	0.12	1.27	DBU	0.49	5.60	0.44	0.50
TSEMO	13	7.82	100.68	0.10	1.19	TBD	1.55	30.27	0.11	0.20
TSEMO	14	4.05	98.57	0.10	0.85	MeTBD	1.04	1.99	1.00	0.50
TSEMO	15	6.25	63.38	0.15	0.92	TBD	1.56	83.66	0.03	0.08
TSEMO	16	6.41	77.30	0.12	1.05	TBD	1.70	37.17	0.08	0.18
TSEMO	17	8.66	46.16	0.19	0.50	TBD	1.40	89.23	0.04	0.12
TSEMO	18	6.87	64.98	0.18	0.88	TBD	2.42	47.28	0.05	0.22
TSEMO	19	8.51	77.41	0.20	0.80	TBD	1.20	60.99	0.04	0.09
TSEMO	20	13.73	37.22	0.10	0.77	TBD	1.09	47.36	0.09	0.11
TSEMO	21	7.75	68.30	0.17	0.97	TBD	0.61	43.68	0.05	0.06
TSEMO	22	8.16	110.00	0.11	0.79	TBD	2.45	24.72	0.17	0.49

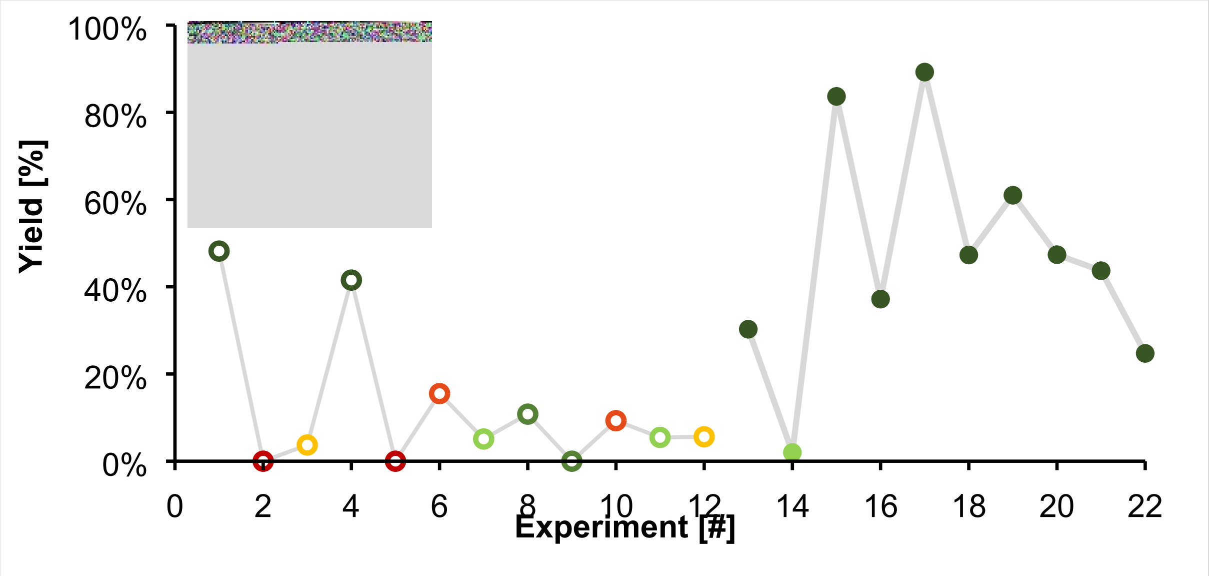

*Figure 22 Multi-objective results of Reaction **2** with semi-continuous encoded catalysts, unfilled circles denote LHC initialization*

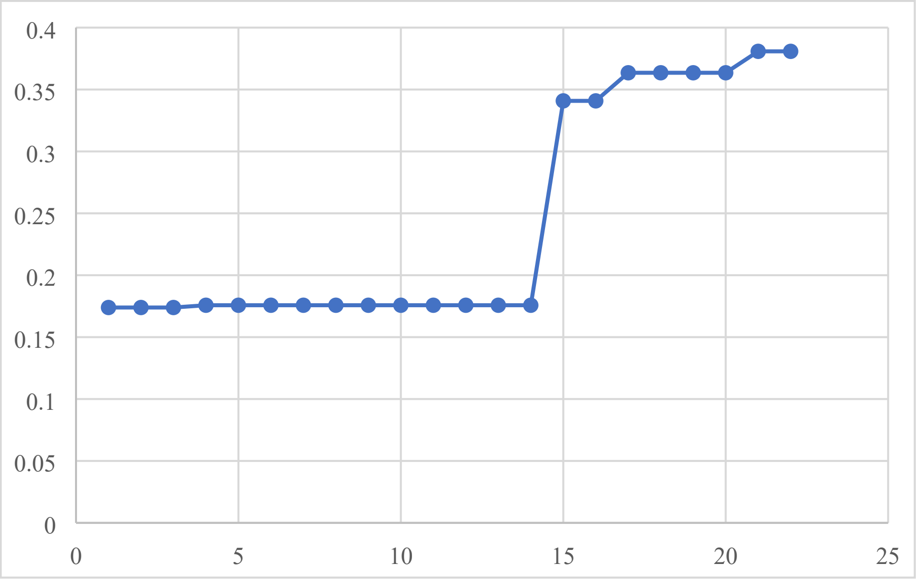

Figure 23 Improvement of hypervolume for reaction 2 with semi-continuous encoded catalysts

7. Transfer to Continuous Flow

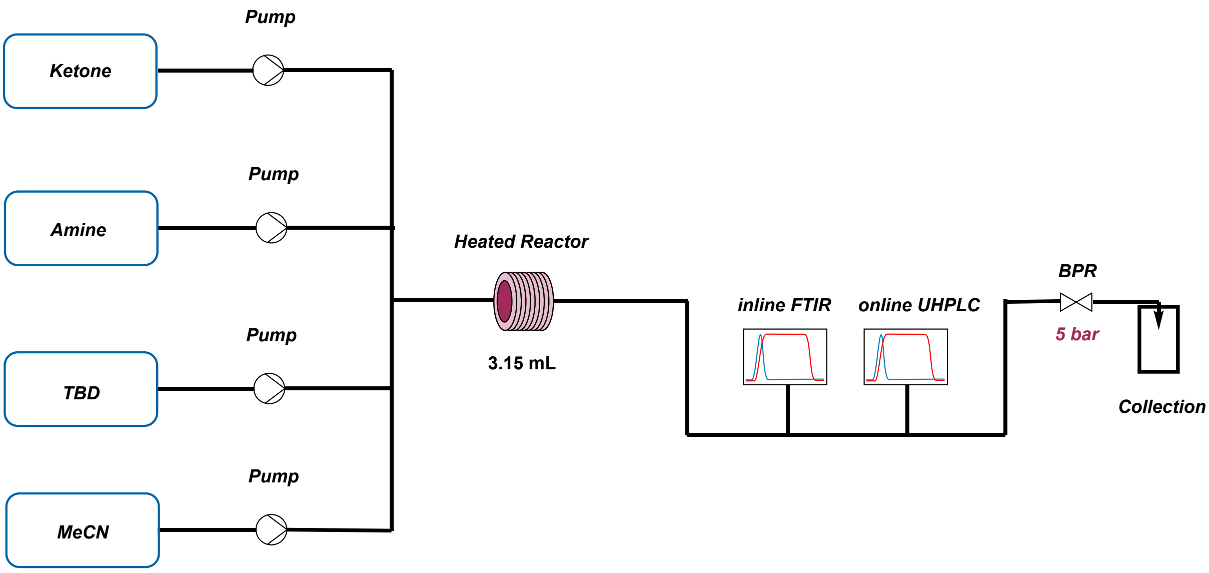

Figure 24 Detailed flow Setup for the continuous validation

The continuous flow validation experiment was carried out in the same coil reactor consisting of 1/16” PFA tubing, submerged in the oil bath of a Thermostat (Huber, Ministat240). The feeds were delivered continuously using four Knauer AZURA P 4.1S HPLC pumps. The streams were combined using a 7-way mixer (IDEX P-151, 83 µL i.V.). After passing the heated coil reactor, the process stream was transported to the FTIR. After the FTIR, the process stream was flowed into the injection valve of the UHPLC. The stream was continuously sampled until the conclusion of the experiment. After passing the UHPLC, the process stream was passed through to a membrane-based BPR (Zaiput, BPR-10, set to 5 bar) in 1/16” PFA tubing and collected after passing through the BPR.

The continuous flow run was performed based on the result of the multi-objective self-optimization. Parameters and result shown below (Table 15).

Table 15 Parameters and result for continuous flow run

RT (min)	T (°C)	Conc SM (mol/L)	Ratio R1/SM	Base Type	Ratio Base	Expected Yield (%)	Yield (%)
6.25	63.38	0,15	0.92	TBD	1.56	83.66	74.20

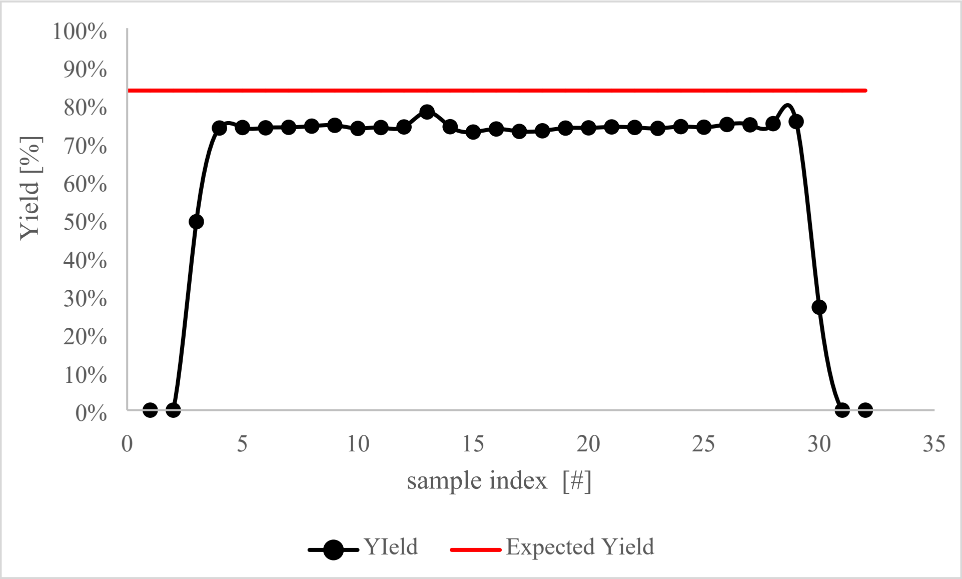

Figure 25 Product yield as determined by UHPLC and expected yield using the conditions from the multi-objective self-optimization

8. Batch Synthesis of Products

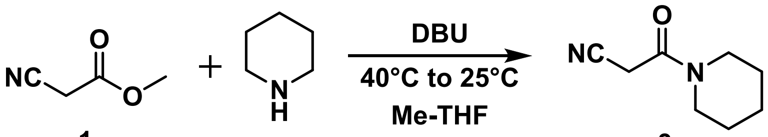

1 2 3

For use in UHPLC calibration, product **3** was synthesized:

A 25 mL round bottom flask was fitted with a magnetic stirring bar and charged with 10 mL Me-THF. **1** (650 µL, 7.3 mmol) was added, followed by **2** (650µL, 6.6 mmol). DBU (500 µL, 3.3 mmol, 0.5 eq) was added dropwise. The reaction was heated to 40°C and stirred for 2 hours and 45 minutes. The reaction mixture was cooled to room temperature. The mixture was diluted with 10 mL ethyl acetate and transferred to a separatory funnel where it was extracted with water (3 x 20 mL), 2 M HCl (1 x 10 mL) and brine (1 x 10 mL). The organic phase was dried over Na_2SO_4 and the solvent evaporated under reduced pressure. The remaining solid was recrystallized in acetonitrile to obtain the product (**3**, 0.75 g, 4.9 mmol, 75% yield).

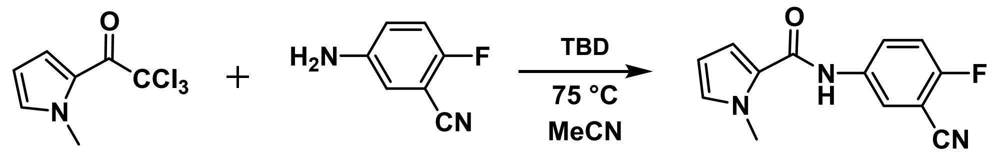

4 5 6

Purification of starting material **4**, the following procedure was applied:

A solvent mixture of cyclohexane and ethyl acetate (150 mL + 50 mL) was prepared. 2 g of crude **4** was dissolved in the solvent mixture (10 mL) and filtered through a silica plug. The filtrate was transferred into a 250 mL round bottom flask and the solvent evaporated under reduced pressure. The remaining solid was used without further purification.

For use in UHPLC calibration, product **6** was synthesized[4]:

A 50 mL round bottom flask was fitted with a magnetic stirring bar and charged with trichloromethyl ketone (**4**, 2.0 g, 8.8 mmol, 1 eq.) in acetonitrile (5 mL). Amine (**5**, 1.5 g, 11 mmol, 1.25 eq.) was dissolved in acetonitrile (5 mL) and added to the flask. TBD (0.5 g, 3.6 mmol, 0.4 eq.) was dissolved in acetonitrile (5 mL) and added slowly. A septum with a balloon filled with argon was put on the flask and the mixture was heated to 50°C and stirred for 1.5 hours. The mixture was allowed to cool to room temperature and 250 µL 6 M HCl were added dropwise. The formed precipitate was filtered and washed with water (3 x 10 mL), affording the product (**6**, 0.99 g, 4.1 mmol, purity 98%, 46.6 % yield).

[1] K. C. Felton, J. G. Rittig, A. A. Lapkin, *Chemistry–Methods* **2021**, *1*, 116–122.

[2] P. Sagmeister, L. Melnizky, J. D. Williams, C. O. Kappe, *Chem. Sci.* **2024**, *15*, 12523–12533.

[3] M. Breugst, F. Corralbautista, H. Mayr, *Chem. - A Eur. J.* **2012**, *18*, 127–137.

[4] F. Medina, W. M. Maton, J. P. Bongartz, D. Kossler, M. Eriksson, J. Weerts, M. Peeters, K. Wegsteen, E. Keppens, *Org. Process Res. Dev.* **2024**, DOI 10.1021/acs.oprd.4c00154.
